# Study of *MDM2* as Prognostic Biomarker in Brain-LGG Cancer and Bioactive Phytochemicals Inhibit the p53-MDM2 Pathway: A Computational Drug Development Approach

**DOI:** 10.3390/molecules28072977

**Published:** 2023-03-27

**Authors:** Partha Biswas, Shabana Bibi, Qudsia Yousafi, Asim Mehmood, Shahzad Saleem, Awais Ihsan, Dipta Dey, Md. Nazmul Hasan Zilani, Md. Nazmul Hasan, Rasha Saleem, Aeshah A. Awaji, Usama A. Fahmy, Mohamed M. Abdel-Daim

**Affiliations:** 1 Department of Genetic Engineering and Biotechnology, Faculty of Biological Science and Technology, Jashore University of Science and Technology, Jashore 7408, Bangladesh; 2 Laboratory of Pharmaceutical Biotechnology and Bioinformatics, Department of Genetic Engineering and Biotechnology, Jashore University of Science and Technology, Jashore 7408, Bangladesh; 3 ABEx Bio-Research Center, East Azampur, Dhaka 1230, Bangladesh; 4 Department of Biosciences, Shifa Tameer-e-Millat University, Islamabad 41000, Pakistan; 5 Yunnan Herbal Laboratory, College of Ecology and Environmental Sciences, Yunnan University, Kunming 650091, China; 6 Department of Biosciences, Sahiwal Campus, COMSATS University Islamabad, Sahiwal 57000, Pakistan; 7 Biochemistry and Molecular Biology Department, Life Science Faculty, Bangabandhu Sheikh Mujibur Rahman Science and Technology University, Gopalgonj 8100, Bangladesh; 8 Department of Pharmacy, Faculty of Biological Science and Technology, Jashore University of Science and Technology, Jashore 7408, Bangladesh; 9 Department of Laboratory Medicine, Faculty of Applied Medical Sciences, Albaha University, Al Bahah 65431, Saudi Arabia; 10 Department of Biology, Faculty of Science, University College of Taymaa, University of Tabuk, Tabuk 71491, Saudi Arabia; 11 Department of Pharmaceutics, Faculty of Pharmacy, King Abdulaziz University, Jeddah 21589, Saudi Arabia; 12 Department of Pharmaceutical Sciences, Pharmacy Program, Batterjee Medical College, P.O. Box 6231, Jeddah 21442, Saudi Arabia; 13 Pharmacology Department, Faculty of Veterinary Medicine, Suez Canal University, Ismailia 41522, Egypt

**Keywords:** *p53* gene, *MDM2* gene, onco-informatics, brain lower-grade glioma (LGG) cancer, predictive biomarker

## Abstract

An evaluation of the expression and predictive significance of the *MDM2* gene in brain lower-grade glioma (LGG) cancer was carried out using onco-informatics pipelines. Several transcriptome servers were used to measure the differential expression of the targeted *MDM2* gene and search mutations and copy number variations. GENT2, Gene Expression Profiling Interactive Analysis, Onco-Lnc, and PrognoScan were used to figure out the survival rate of LGG cancer patients. The protein–protein interaction networks between *MDM2* gene and its co-expressed genes were constructed by Gene-MANIA tool. Identified bioactive phytochemicals were evaluated through molecular docking using Schrödinger Suite Software, with the MDM2 (PDB ID: 1RV1) target. Protein–ligand interactions were observed with key residues of the macromolecular target. A molecular dynamics simulation of the novel bioactive compounds with the targeted protein was performed. Phytochemicals targeting MDM2 protein, such as Taxifolin and (-)-Epicatechin, have been shown with more highly stable results as compared to the control drug, and hence, concluded that phytochemicals with bioactive potential might be alternative therapeutic options for the management of LGG patients. Our once informatics-based designed pipeline has indicated that the *MDM2* gene may have been a predictive biomarker for LGG cancer and selected phytochemicals possessed outstanding interaction results within the macromolecular target’s active site after utilizing in silico approaches. In vitro and in vivo experiments are recommended to confirm these outcomes.

## 1. Introduction

The protein 53 (p53) was first found in 1979 and is known as “Guardian of the Genome” for controlling cell division and stopping tumor formation [1]. The P53 protein is a transcription factor regulating the expression of a wide effective range of genes that play a role in apoptosis, cell cycle regulation, differentiation, and DNA repair [2]. It also plays a pivotal role in starting a cell protection program in the response to intracellular stresses. Hence, *p53* mediated diverse signal transduction networks engaged in the cell’s defense against cancer. Notably, intrinsic and extrinsic stress signals affecting cellular homeostatic mechanisms may activate the *p53*-directed pathway/s [3,4]. Conversely, *p53* can induce apoptosis, prime DNA repair, or a DNA replication mechanism by following in a transcription-independent manner [5,6]. Actually, the expression of *P53* is low in normal conditions, whereas damage to the DNA of cells may elevate the expression pattern of the P53 protein [7,8]. The p53 protein loses its ability through interacting with MDM2, a negative regulator of p53 protein expression [9]. In higher eukaryotes, two closely related proteins named MDM2 (also known as HDM2 for its human homolog) [10] and MDMX (also known as MDM4) [11] closely monitor p53. MDM2 and MDMX primarily carry out their carcinogenic activity by negatively regulating the p53 protein’s stability and activity in a feedback loop, as they collaborate to inhibit p53’s transcriptional activity [10,12]. The structure of MDM2 is like a hydrophobic groove that is made up of a loop and two helices. The back of the groove is made up of two sheet structures. The structural part that links with P53 sites is made up of the key residues: Leu54, Leu57, Gly58, Ile61, Met62, Tyr67, Gln72, His73, Val75, Phe91, Val93, His96, Ile99, and Tyr100 [13,14,15]. The *MDM2* gene is found on chromosomes 3q15 and 12q14 and it has a total length of 491 amino acid residues, and it is primarily regulating *p53* stability through ubiquitination, which targets the tumor-suppressor protein for unregulated proteasome degradation, whereas *MDMX* primarily operates as a major *p53* transcriptional antagonist independent of *MDM2* in the absence of its expression [16]. Simultaneous disruption of the *p53-MDM2/MDMX* linkages results in prolonged and robust *p53* activation, implying a possible anticancer approach [17,18,19]. Recently discovered is that *MDM2* has been found to disrupt the function of *p53* via blocking *p53* transcriptional activity and p53 protein degradation; such inconsistent activity of *MDM2* for suppression of *p53* expression is related to the higher progression of various types of malignancies, especially brain cancer [20,21,22]. It is important to note that *MDM2* overexpression is linked to poor survival, for breast cancer, hepatocellular carcinomas, and brain lower-grade glioma’s patients [23,24,25,26]. To date, many drugs have been found on the market, but most of them have fewer and more side effects. Further, plant-derived compounds are safer and effectively work towards brain cancer.

There has been an excellent intention for discovering cancer therapeutics from bioactive plant sources because anticancer drugs that are derived from plant sources are less expensive than synthetic cancer therapeutics [27,28]. Medicinal plants have fewer toxic effects on human health than synthetic products [29]. Several plant-derived substances, including Vinblastine [30], Taxol [31], and Topotecan [32], etc., are used as cancer therapeutics in clinical research. Because of recent advances in genomics and proteomics, determining the target prospective phytocompounds has exhibited effective anticancer potential in several clinical cancer research studies [33,34]. However, the discovery of new anticancer drugs has become an urgent requirement in modern times because of raising the drug’s resistance properties to cancer cells. In contrast, bioactive phytochemicals or plant-derived compounds have significantly positive outcomes in several clinical trials [35]. Several well-known phytochemicals have been discovered to possess anticancer characteristics in colorectal, prostate, breast, and glioblastomas [36,37]. Phytochemicals possessed potential anti-proliferative effects in cancer tissues via modulating several of the cancer’s cellular signaling mechanisms [38,39,40].

In this current in silico study, firstly, we have conducted in-depth research to explore the nature and clinical relationship of the *p53* and *MDM2* gene in the formation of brain lower-grade glioma (LGG) cancer by using several cancer data sets and suggested that the *MDM2* gene can be a prognostic biomarker for the early stage LGG cancer. Additionally, to overcome the disease’s severity, we utilized the computational drug design methods, which suggested some natural flavonoid-derived compounds which have potential anticancer properties, and also checked its ADMET properties, because of the positive outcomes from the ADMET results following the molecular docking with the MDM2 protein (PDB ID-1RV1). Afterward, the best-selected phytochemicals were applied for post-docking analysis and molecular dynamic simulation (MDS) study to evaluate their potential for inhibiting the overexpression of MDM2 protein.

## 2. Results

### 2.1. MDM2 Gene Expression Analysis

**Figure 1:**
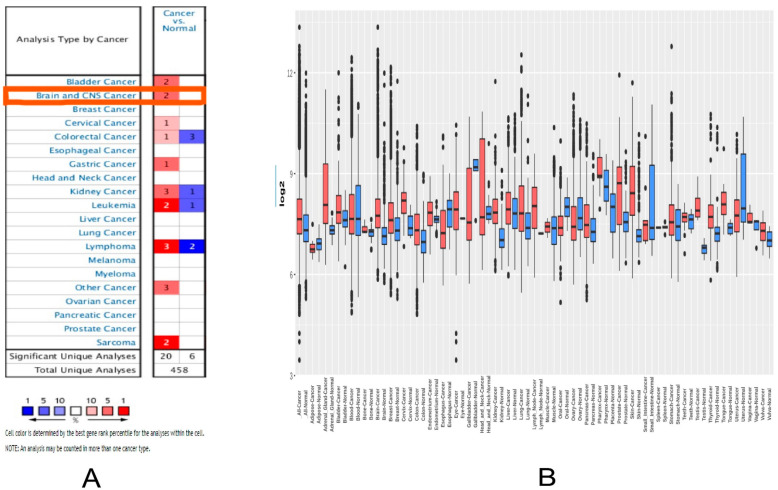
The expression profile of the *MDM2* gene mRNA in a variety of cancer types. (**A**) The mRNA expression of *MDM2* in various malignancies from the Oncomine database, with red cells denoting datasets with strong mRNA upregulation and blue cells denoting datasets with notable mRNA downregulation. (**B**) Analysis of *MDM2* gene pan-cancer expression profiles from the TIMER database in dot plot where each dot represents different sampling expression. The expression of mRNA in different cancer types is measured in transcripts per million (TPM).

First, the Oncomine, GENT2, and GEPIA2 databases were used to evaluate the patterns of the differentially expressed *MDM2* gene in distinct cancer types. *MDM2* mRNA expression patterns were identified to be significantly different in several malignancies when they are compared with the standard (Figure 1A). There was a total of 12 Oncomine brain cancer databases, and the statistical data on the *MDM2* gene expression are provided in “Supplementary Materials Table S1” in various Oncomine CRC subtypes. The *MDM2* gene, according to the GEPIA2 database, was substantially elevated in brain and CNS cancer compared to matched normal tissues (Figure 1).

**Figure 2:**
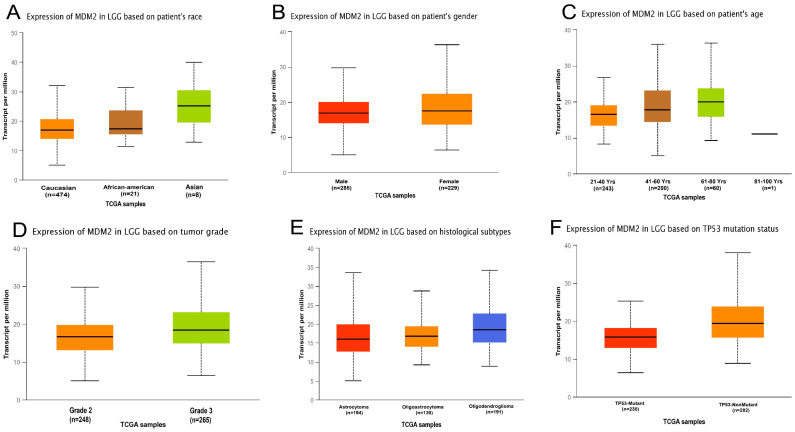
Using the UALCAN database, we analyzed the prognostic significance of the *MDM2* gene in TCGA LGG, comparing cancerous and normal stages depending on several characteristics. (**A**) The expression of *MDM2* in LGG varies depending on the patient’s race. (**B**) The expression of *MDM2* in LGG is dependent on the gender of the patient. (**C**) The expression of *MDM2* in LGG is dependent on the patient’s age. (**D**) The expression of *MDM2* in LGG depends on the tumor grade. (**E**) The expression of *MDM2* gene in LGG cancer based on histological subtypes. (**F**) The expression of *MDM2* in LGG based on the TP53 gene mutation status.

Moreover, the *MDM2* gene was found to be considerably elevated in various cancers, including invasive bladder cancer, kidney cancer, leukemia, lymphoma, and sarcoma (Figure 1B). However, the *MDM2* gene was suppressed in several types of cancer, including cervical, colorectal, and stomach (Figure 1B). In addition to this, the results obtained from the Oncomine and GEPIA2 databases were validated by comparing them to those obtained from the GENT2 database. It also displayed a boxplot of the *MDM2* gene expression profile in 72 different connected normal and cancer tissues, which demonstrated that this gene was increased in numerous types of cancer, including brain cancer, and that this was true for numerous types of cancer. Furthermore, the mRNA expression of the *MDM2* gene in brain tissues was compared to normal samples using different clinicopathological limits from the UALCAN and GEPIA2 databases. According to the UALCAN database, the amount of mRNA produced by the *MDM2* gene was shown to be significantly higher in TCGA brain cancer samples. Several clinicopathological parameters, including sample type, cancer stage, ethnicity, gender, body mass index, age, histopathologic subtype, and *TP53* mutation status were found to correlate highly with *MDM2* gene overexpression in “TCGA” brain tissues, as measured by mRNA expression. The data are depicted in Figure 2 and Supplementary Materials Table S2.

**Figure 3:**
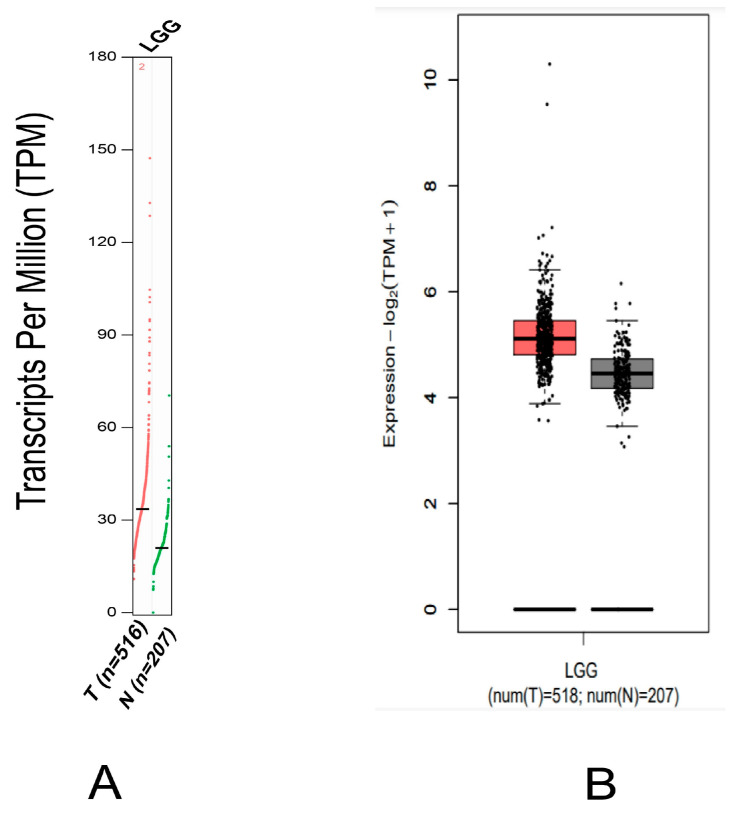
*MDM2* gene expression profile by tissue in LGG derived from the GEPIA2 database, where T denotes the tumor tissue and N is depicted for the normal tissue. (**A**) *MDM2* gene expression in LGG, depicted as a box plot with the red box representing tumor samples and the black box representing normal samples. (**B**) GEPIA2 database was also used to compare the transcription pattern of *MDM2* in brain lower-grade glioma (LGG) patients with their respective normal samples.

Likewise, *MDM2* gene overexpression was investigated in several clinical phases of brain cancer (Figure 3B). Thirdly, the UALCAN database was utilized to assess the *MDM2* gene promoter methylation level in brain cancer, where the methylation status is exhibited as a beta value scale. Promoter DNA that has been methylated is shown by a beta value that ranges from 0 (that means completely unmethylated) to 1 (that means highly methylated).

**Figure 4:**
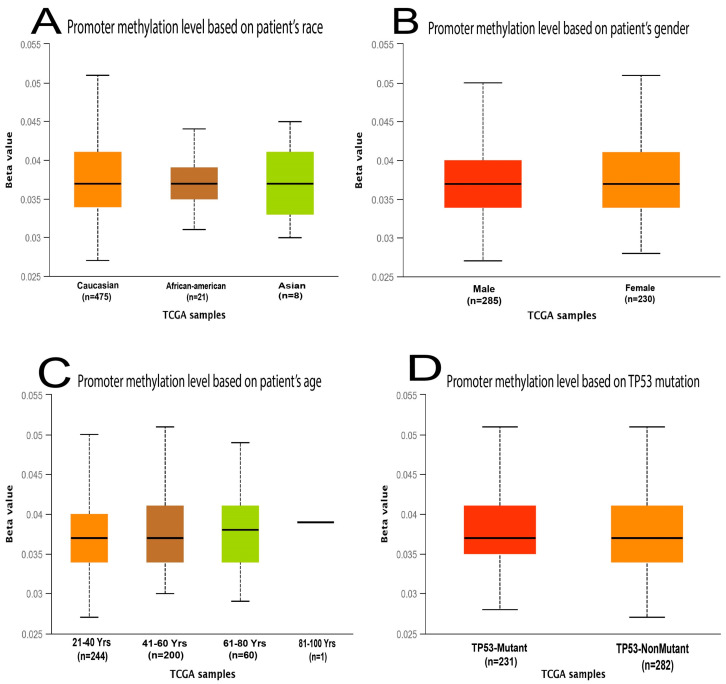
The UALCAN database was used to determine the promoter methylation status of the *MDM2* gene for LGG cancer depending on multiple variables. (**A**) The extent to which *MDM2’s* promoter is methylated in LGG is determined by the patient’s race. (**B**) The extent to which *MDM2’s* promoter is methylated in LGG is determined by the patient’s gender. (**C**) *MDM2* promoter methylation levels in LGG are determined by the patient’s age. (**D**) *MDM2* promoter methylation level in LGG as a function of *TP53* mutant status. The beta value, according to UALCAN, shows the degree of DNA methylation, ranging from 0 (that means completely unmethylated) to 1 (that means completely methylated). The bold *p*-value shows the statistical significance.

The rate of promoter methylation in *MDM2* gene was greater in brain cancer compared to the standard tissues based on sample type, different cancers stages, ethnic background, sex, weight, age, histopathologic subtypes, and *TP53* mutation status. The level of methylation of *MDM2’s* promoter in LGG was determined and exhibited in Figure 4 and Supplementary Table S3.

### 2.2. Genetic Mutations and Copy Number Alterations (CNAs) Analysis of MDM2 Genomic Sequences Correlated with Brain Cancer Development

We collected different genetic alteration data utilizing the cBioPortal server in order to investigate the significance of the *MDM2* gene in the progression of LGG Cancer. To begin, we queried this database for changes in *MDM2* expression using 5408 samples from 5208 LGG cancer patients across 9 studies (Table 1).

**Figure 5:**
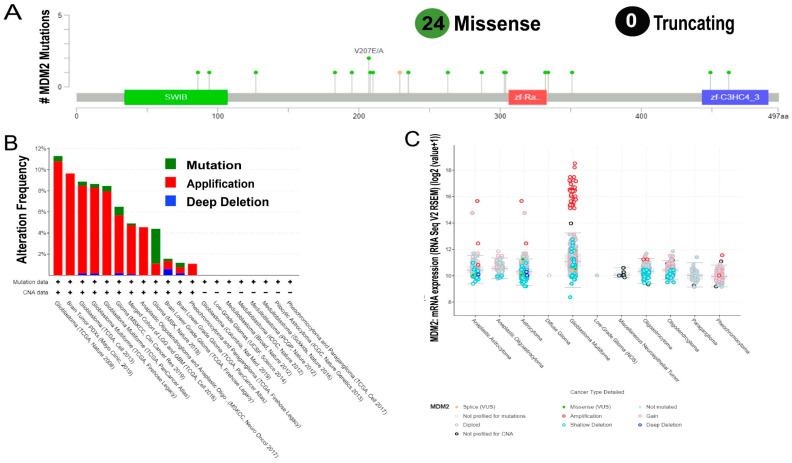
Representation of the genetic alterations in *MDM2* protein sequences because of LGG progression enhancement. (**A**) A lollipop plot was used to identify 28 mutation variants in the *MDM2* protein sequence. (**B**) In a bar diagram, two types of *MDM2* modification frequencies were presented across distinct LGG research. (**C**) *MDM2* expression levels were examined in relation to several classifications of genetic changes using graphical plots based on the RNA seq V2 relative standard deviation of the mean scale (RNA seq V2 RSEM).

These 28 mutations were shown in a lollipop plot, with 24 representing missense and null truncating (Figure 5A). Following that, the genetic alteration appearance of the *MDM2* gene was examined utilizing data from several brain cancer studies. Based on our research findings, it was observed that the rate of *MDM2* change varied considerably among various LGG investigations. Among these investigations, *MDM2* was primarily changed in glioblastoma, with the highest incidence of modification at 11.90%. LGG studies, on the other hand, also had the lowest rate of change (Figure 5B). Finally, a distinctive transcriptional analysis was performed between the expression of *MDM2* mRNA and probable CNAs. The amount of *MDM2* mRNA expression was found in this investigation, and the expression was the most overexpressed CNA on the RNA seq V2 RSEM scale.

Shallow deletion, on the contrary, was the most reported change based on expression frequency (Figure 5C). Thus, it was clear that different genetic changes in *MDM2* resulted in LGG formation.

### 2.3. The Analysis of Prognostic Value and Survival Assay of MDM2 Gene

**Figure 6:**
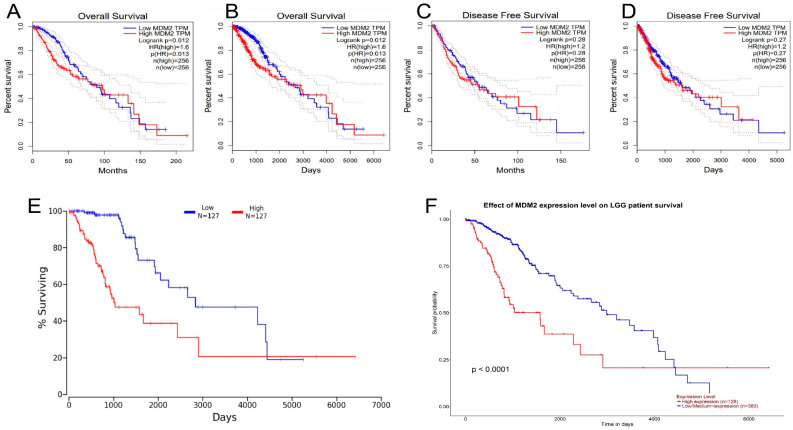
The analysis of survival assay for *MDM2* gene in brain lower-grade glioma cancer (LGG). The analysis of survival plots has been depicted from GEPIA, Onco-Lnc, and UALCAN servers. Here, the plots were (**A**) survival rate (overall) per month, (**B**) survival rate (overall) per day, (**C**) diseases-free survival per month, (**D**) diseases-free survival per day, (**E**) survival plot collected from Onco-Lnc database, (**F**) survival data from UALCAN server.

Multiple online resources, namely, GEPIA, Onco-Lnc, GENT2, and UALCAN databases, were used to evaluate the expression of *MDM2* gene and the outcomes of clinical prognosis of the patients with LGG greater details. The GEPIA database provides survival graphs for disease-free survival and overall survival. The 50 percent median value, 95 percent interval of confidence at the same time GEPIA server applied for the determination of a hazard ratio to create the survival charts. In the database, *MDM2* lower expression was also associated with a higher OS and DFS (Figure 6A–D). Both the lower and upper percentiles were set to 25 in order to create an updated plot as Kaplan–Meier by evaluating a broad range of LGG disease research through the Onco-Lnc server. The OS data were then matched from the UALCAN server, where analysis of the gene expression was performed, along with utilizing the LGG patient’s survival statistics. Onco-Lnc explored 127 cancer samples with high and low expression of *MDM2* gene.

**Figure 7:**
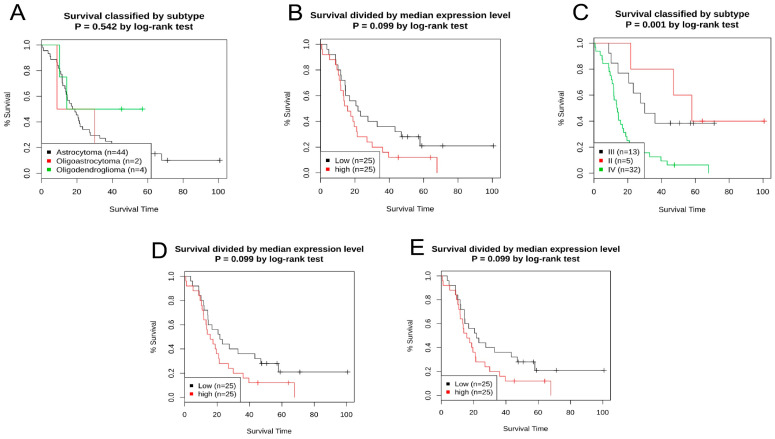
Prognostic significance of the *MDM2* gene in human brain lower-grade glioma (LGG) cancer as determined by the GENT2 server. (**A**) Overall survival for the disease stage was determined by dividing KM plots by the median cutoff. (**B**) Overall survival for the disease stage was determined by dividing the KM plots by the median cutoff. (**C**) Overall survival grade in KM plots was divided by subgroups. (**D**) Survival (overall) for grades KM plots separated via median value. (**E**) Survival (overall) for histology is determined for adding KM plots by the median cutoff.

UALCAN tool investigated the 128 LGG cancer patient samples and identified the higher expression level, as well as 383 samples with lower expression, in order to determine which samples were more prevalent. According to the Onco-Lnc and UALCAN databases’ graphs, patients with low *MDM2* expression had a better prognosis (Figure 6E,F). Kaplan–Meier curve was generated using subgroups and a median cutoff value from the GENT2 database, where colon tissues were classified according to molecular subtype, Dukes stage, AJCC stage, and tissue histology. Low and high *MDM2* expression levels had similar survival rates (Figure 7). Overexpression of the *MDM2* gene was found to be related to a lower prognosis for patients with brain lower-grade glioma (LGG). Consequently, it can be determined that *MDM2* is a tumor prognostic gene for LGG after studying its prognostic value and survival time.

### 2.4. Study of Correlated Genes, and PIP Network

Two web-based databases, GEPIA and UA2CAN, were utilized to find genes associated with *MDM2* for LGG. The Pearson correlation co-efficient (CC) value > 0.47 is regarded as quite a significant value. YEATS4 is found to have the most positive connection with *MDM2* in LGG. To create an interaction network, twenty related genes were retrieved from both websites. The network was investigated using the Gene MANIA online tool. Twenty (*TCAP*, *CDKN2A*, *PIAS2*, *MTBP*, *TP53*, *CCNG1*, *GLIS2*, *TP538P1*, *APEX1*, *RPL11*, *IGF1R*, *PHOSPHO1*, *PJA1*, *AKAP5*, *MDM4*, *EPB41L1*, *DLG1*, *USP15*, *RPL5*, *DLG4*) associated comparable genes, as well as *MDM2*, were used to construct an interaction network (Supplementary Materials Figure S1).

**Figure 8:**
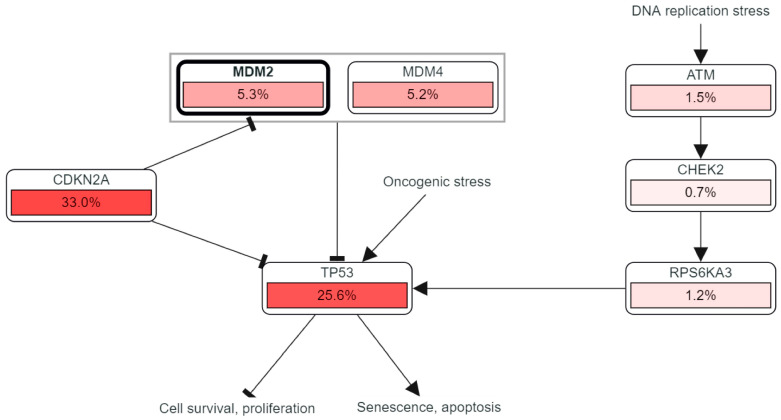
The interaction pathway of *MDM2* with its correlated genes which are significantly associated with the progression of brain lower-grade glioma (LGG) cancer. The pathway has been depicted from cBioportal server after securing their cordial permission.

A PPI (preparing interaction pathway) network demonstrated 3.03% co-localization and 8.01% co-expression, which were constructed by an automatically selected weighting mechanism. The gene cBioPortal database and MANIA server were utilized for the evaluation of the PPI network of the *MDM2* gene to express its link with strongly correlated genres (Figure 8). Various variables, including co-localization, co-expression, pathway, shared protein domains, physical relationship, genetic interaction, and anticipation were used to establish an automatically generated weighting mechanism for the network of twenty (*TCAP*, *CDKN2A*, *PIAS2*, *MTBP*, *TP53*, *CCNG1*, *GLIS2*, *TP538P1*, *APEX1*, *RPL11*, *IGF1R*, *PHOSPHO1*, *PJA1*, *AKAP5*, *MDM4*, *EPB41L1*, *DLG1*, *USP15*, *RPL5*, *DLG4*) genes connected to *MDM2*. The interaction network demonstrated 77.64% physical interaction, 8.01% co-expression, 5.37% predicted, 3.63% co-localization, 2.8% genetic interaction, 1.88% pathway, and 0.60% associated protein domains (Supplementary Materials Figure S2).

### 2.5. ADMET Profiling

The analysis and optimization of the pharmacokinetic features of the chosen therapeutic agents were performed using Swiss ADME and pKCSM web-based programs. The flavonoid derivatives Taxifolin, (-)-Epicatechin, and Galangin have molecular weights of 304.25, 290.27, and 270.24 gm/mol, respectively. All the substances had the same bioavailability score (0.55), no AMES toxicity, and the lowest Lipinski rule violations (0). When compared to the reference medications, the molecules have the smallest topological polar surface area. It should also be mentioned that the chemicals had no hepatotoxic effects. While the value range between 0.300–0.450 in humans was identified as the maximum tolerated dose (log mg/kg/day), acute oral rat toxicity (LD50) for the flavonoid compounds was found to be between the ranges of 2.100–2.500. In addition, the total clearance (ToC) ranged from −0.050 to 0.300 (log mL/min/kg), and the projected octanol/water partition coefficient (Log P) fell between 0.50 and 2.00 for all substances. The permeable value of the blood-brain barrier (BBB) is −0.70 to −1.00; additionally, Table 2 precisely exhibits hydrogen bond donor/s, hydrogen bond acceptor/s, rotatable bond/s, and so forth.

### 2.6. Active Site Identification and Generation of Receptor Grid

After analyzing the MDM2 protein structure (PDB ID: 1RV1), five predicted active sites (ASs) were found (AS1, AS2, AS3, AS4, and AS5) along with the analysis of protein’s crystal and co-crystal structures. A hydrogen-bonding acceptor map, a hydrogen-bonding donor map, a hydrophobic map, and a hydrophilic map are displayed in each and every predictive AS. As a result, an oxygen atom will also accept the hydrogen bond from the receiver if it is located in a metal binding zone, and it will also be associated with a metal center. Polar hydrogen on amide nitrogen was produced in the donor region [41]. Site Score examines the ligand-binding prime of a site and correctly classifies prospective binding sites to remove those likely to be un-relevant to the pharmaceuticals and determines the drug-ability of a binding site through the D-score [42]. By combining Site Score and D-Score, we identified five potential binding sites. The most promising of the binding sites for MDM2 residues is designated as AS1, which can be predictive. Glide is looking for the advantageous relationships between ligand and the receptor molecules (Grid-based Ligand Docking with Energetics). The shape and features of the receptor are shown on a grid by several field sets that gradually yield increasingly accurate ligand measurements [43]. For molecular docking experiments, the AS1 grid was generated using a receptor grid generating program (scaling factor of 1.0 and partial charge cut of 0.25) from all the predicted structure.

### 2.7. Interpretation of Molecular Docking

Selected flavonoid phytochemicals that interact with the MDM2 protein were identified by a molecular docking method carried out utilizing the Maestro package platform. Between the macromolecules and ligands, the Maestro application generated the highest possible docking score. As a control ligand, Temozolomide (control) and Imidazoline (the native ligand of 1RV1) were included in this study, and it acquired −6.5 Kcal/mol and −2.5 Kcal/mol binding affinity, respectively. The aromatic ligand Taxifolin performed the best fitting score of −10.0 Kcal/mol, and the other potential bioactive phytochemicals, such as (-)-Epicatechin, and Galangin exhibited the best docking value of −8.8 and −7.4 Kcal/mol, comparatively, which are represented in Table 3.

### 2.8. Visualization of Post-Docking Protein-Ligands Interactions

**Figure 9:**
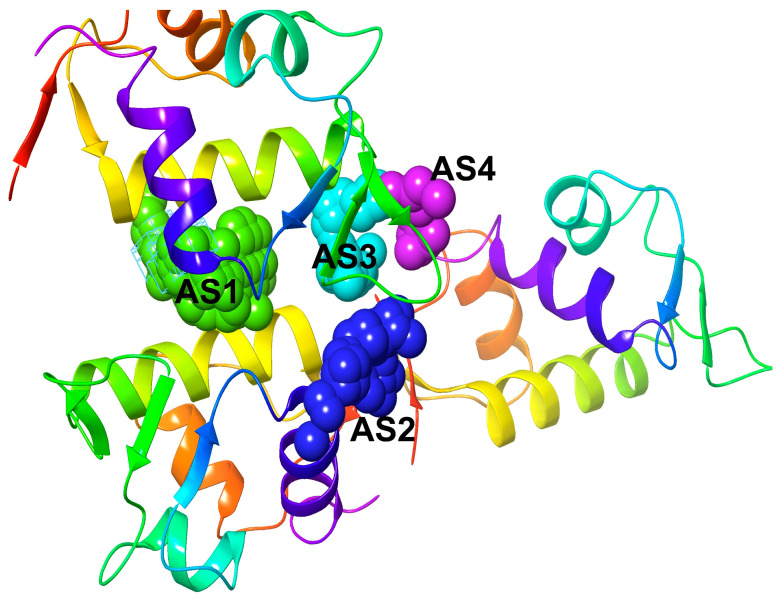
Representing the predictive active site (AS) of MDM2 protein retrieved from Site Map application of Maestro package, Schrödinger Software, where the ball and sticky shape with different colors possessed these predictive active sites with the receptor atom (signified by the interactive amino acid residue numbers of the MDM2 protein).

**Figure 10:**
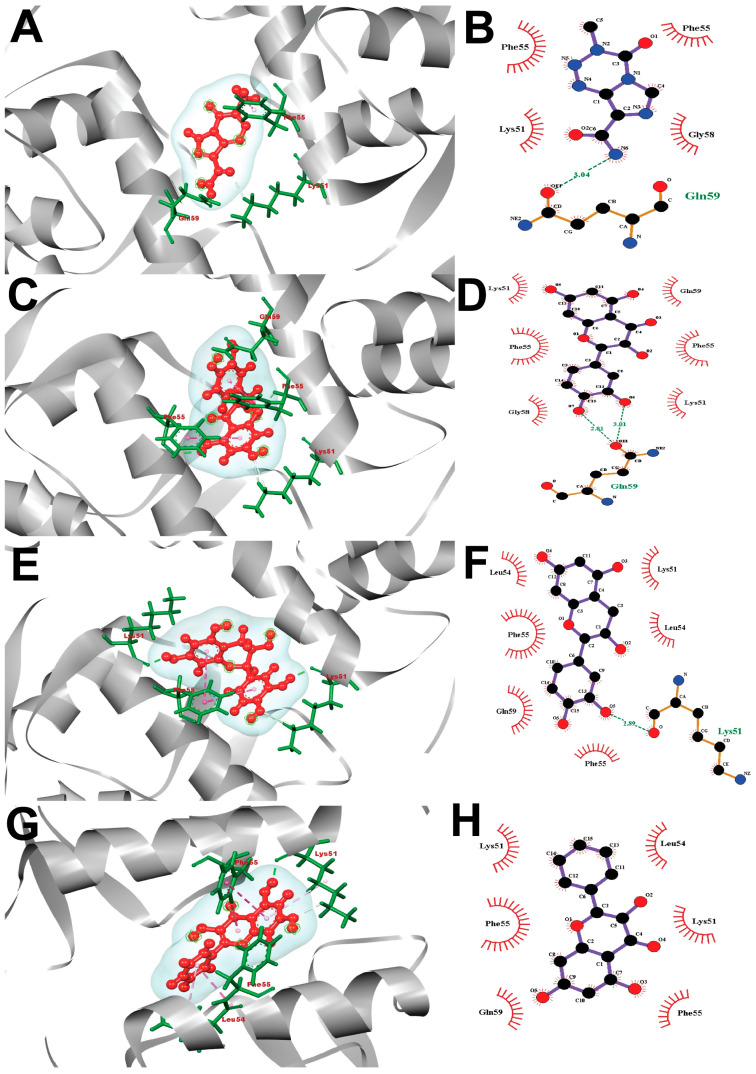
The interaction of all the ligand compounds with MDM2 protein was exhibited. The left-sided figures illustrate the 3D complex, while the right-sided figures illustrate the 2D complex of the protein–ligand interaction. Here, (**A**,**B**) represented the 3D and 2D interaction of control drug Temozolomide (PubChem CID-5394) with MDM2 protein, whereas (**C**,**D**)-compound Taxifolin (PubChem CID-439533), (**E**,**F**)-compound (-)-Epicatechin (PubChem CID-72276), and (**G**,**H**)-compound Galangin (PubChem CID-5281616) were depicted comparatively in interaction with the MDM2 protein complex.

Observations of the molecular interaction among the selected ligands and the targeted protein were accomplished using the Ligplot+ Version 2.2 and BIOVIA Discovery Studio Visualizer tool. All the docked complexes have been performed in Ligplot+ Version 2.2, and the interactions (mainly hydrophobic, and non-covalent) were calculated in Figure 9 and Figure 10. The “MDM2-Temozolomide (control drug)” complex was fitted by only a single hydrogen bond (with Gln59 (3.04 Å), and four hydrophobic bonds with the Phe55, Phe55, Lys51, Gly58). Additionally, the “MDM2-Taxifolin” complex was stood by two hydrogen bonds (Gln59 (2.81 Å), Gln59 (3.01 Å)), and six hydrophobic bonds (Lys51, Phe55, Gly58, Lys51, Phe55, Gln59), whereas one hydrogen bond (Lys51 (2.89 Å) and six hydrophobic bonds (Leu54, Phe55, Gln59, Phe55, Leu54, Lys51) were exhibited by (-)-Epicatechin against the MDM2 receptor. Galangin stabilized six hydrophobic bonds (Lys51, Phe55, Gln59, Phe55, Lys51, Leu54).

### 2.9. Molecular Dynamics Simulation (MDS) Analysis

Molecular dynamic simulation (MDS) is the process where stability, rigidity, bond interaction and folding of the protein molecule is analyzed in an artificial normal physiological condition. An amount of 100 ns MDS was conducted, and steady characteristics of the protein–ligand complexes, including control, were analyzed. It has been performed based on trajectories such as RMSD, RMSF, Rg, amount of hydrogen bond, and MM-PBSA.

#### 2.9.1. RMSD Analysis

**Figure 11:**
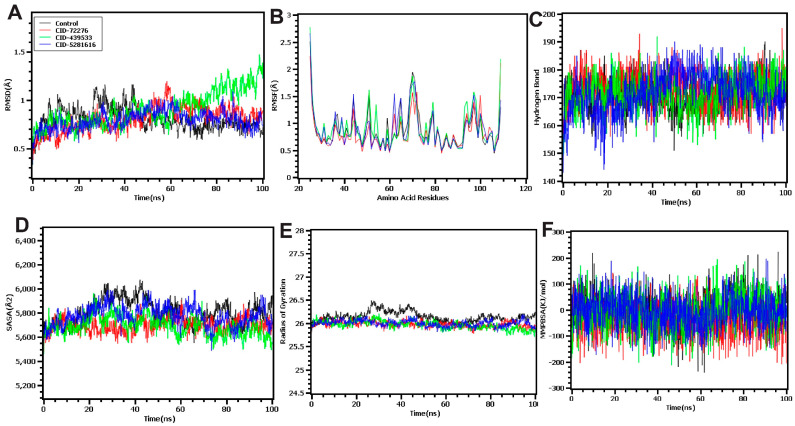
(**A**) The graph showed the RMSD data of all protein–ligand complexes at 100 ns duration of the simulation. (**B**) Diagrammatic illustration of RMSF values of five complexes. (**C**) The graph shows the number of hydrogen bond interactions. (**D**) The diagram showed the SASA values of all complex structures (protein-ligand) at 100 ns. (**E**) The graph shows the Rg values of protein–ligand complexes. (**F**) The chart depicted the importance of MMPBSA of all the protein–ligand complexes at a 100 ns simulation period. All MDS evaluations of the control drug were displayed by ash colour, whereas the compounds CID-439533, CID-72276, and CID-5281616 were shown in green, orange, and blue, respectively.

The root-mean-square deviation, or RMSD, is a metric that reflects the mean value which is altered by the dispersion of atoms from a certain configuration relative to a reference frame and is used to determine whether or not the simulation has reached equilibrium [44]. During the 100 ns simulation time, the fluctuation of the mean values was analyzed, and less fluctuation indicates the better conformational stability of the complex. Higher fluctuation denotes poor strength in the artificial environment (Figure 11A). The compound CID-439533 showed more fluctuation than other compounds after the 60 ns period, whereas the control drug displayed fluctuation slightly at the beginning. The remaining two compounds, such as CID-72276 and CID-5281616, exhibited as much overlapping in fluctuation throughout the 100 ns time. All the complexes showed better mean values, which were within the 0.5–1.2 Å.

#### 2.9.2. RMSF Study

The root mean square fluctuation (RMSF) indicates the steady characteristics of the macromolecule and provides some information about the internal folding changes of the macromolecule along with its chain [45]. The RMSF values of the selected phytochemicals such as CID-72276 (orange), CID-439533 (green), CID-5281616 (blue), and control (grey) have been calculated, as shown in Figure 11B. More fluctuations were found between 45 to 75 amino acid residues, and at 70 amino acid residues, it provided the highest peak of control than other phytochemicals. All the compounds, along with the control, displayed moderate fluctuation between 0.5 Å to 2.8 Å. After 75 amino acid residues, almost all the compounds showed overlapping in their fluctuations with some divergence. Compounds such as CID-439533 (green) and CID-5281616 (blue) displayed more fluctuation than CID-72276 (orange).

#### 2.9.3. Hydrogen Bond Analysis

Hydrogen bond took part in binding the ligand molecule with the targeted receptor and indicates the possibility of drug-likeness and metabolic properties [45]. The interaction of hydrogen bonds with ligands enhances the stability of the complex. All the complexes of this research showed a more stable condition of the hydrogen bond interaction for the 100 ns period mentioned in Figure 11C. Therefore, a more stable hydrogen bond interaction of the complexes indicates rigidity and proper metabolism and absorption in the body.

#### 2.9.4. Analysis of SASA Value

The solvent accessible surface area, also known as the SASA, is an additional trajectory of MD simulation that displays the quantities of changes in protein surface area when subjected to extreme circumstances [46]. Initially, overlapping SASA values were found for 10 ns time after, then they showed some fluctuations (Figure 11D). The compounds such as CID-439533 (blue) and CID-72276 (orange) displayed lower SASA values to determine the more compactness of the targeted protein surface, and these values are steady. On the other hand, control and CID-5281616 (green) displayed the standard value compared to the selected protein–ligand complex.

#### 2.9.5. Study of Rg

The radius of gyration or Rg of the protein–ligand complexes was measured to find out how they moved and how stiff they were [47]. A graphical demonstration was found of Rg values from the following (Figure 11E). Almost all the phytochemicals were found to be more stable and showed the same rigidity with few fluctuations of the protein–ligand complex. In contrast, the control drug displayed moderate fluctuation, and more fluctuation stayed between 25–45 ns, comparatively to other selected ligand compounds. The compound CID-439533 showed more rigidity among all compounds.

#### 2.9.6. Analysis of MM-PBSA Value

From the interpretation of molecular mechanics, Poisson-Boltzmann Surface Area (MM-PBSA) found the free energy in studying protein–ligand complexes. Here, all the selected natural bioactive compounds possessed the control drug’s potential binding free energy value (Figure 11F). Here, the ligands Taxifolin (PubChem CID-439533) and Galangin (PubChem CID-5281616) possessed a higher MMGBSA value than the control drug Temozolomide (PubChem CID-5394).

## 3. Discussion

Several research studies have recently reported that abnormal amplification of the *MDM2* gene in neuroblastoma and glioblastoma causes the protein degradation of the *p53* gene that initiates the high progression of tumor tissue in brain cancer [48]. This study compared *MDM2* gene mRNA expression patterns in different types of cancer samples, particularly in LGG cancer samples, with their comparable normal samples, which were overexpressed in the brain tissue and CNS cancers [49]. In addition, the expression is downregulated in the following cancers including gastric cancer, kidney cancer, bladder cancer, leukemia, sarcoma colorectal cancer, lymphoma, and many more (Figure 1). Moreover, based on a variety of clinicopathological criteria, *MDM2* was found to be considerably elevated in LGG when compared to normal samples (Figure 2 and Figure 3). In this way, there is a significant relationship between higher *MDM2* expression and the potential for the formation of tumor metastasis. Numerous studies have also established the *MDM2* gene’s efficacy as a prognostic biomarker in a variety of cancer types, including human lung carcinomas, cervical carcinogenesis, bladder cancer, neck cancer, and ovarian cancer, as well as glioblastoma [50,51]. As a result, it is proposed that the unregulated expression of the *MDM2* gene relates to the development of LGG. *MDM2* has recently gained attention as an effective therapeutic target due to its clear correlation to cell cycle control and cancer. Consequently, all bioinformatics approaches were carried out in this study using many powerful publicly accessible datasets, revealing that *MDM2* may be an effective prognostic biomarker for brain lower-grade glioma cancer treatment. The phosphorylation or methylation of *MDM2* in the promoter region may be responsible for the different roles in several types of human cancer. To investigate this, we have analyzed the amount of methylation of the *MDM2* gene promoter in LGG using a variety of clinicopathological variables (Figure 4). The frequency of promoter methylation fluctuates drastically between LGG phases, and the level of methylation was effectively higher in tumor samples than in normal ones.

On top of that, 13 cancer studies were used to assess the mutations and CNAs in the MDM2 protein sequence to determine whether the gene has functional relevance in LGG development. Overall, 28 alterations were discovered, with 24 being missense mutations (Figure 5). Additionally, some studies proposed that finding genomic regions that undergo frequent change could be a useful strategy for identifying oncogenes in human cancers [52]. It became clear because of this finding that *MDM2* could play an important function in the formation of LGG. The *MDM2* gene’s prognostic significance for LGG was determined using a KM plotter from following sites GENT2, GEPIA, UALCAN, Onco-Lnc, and survival curve analysis revealed that decreased *MDM2* expression was associated with a greater OS and DFS (Figure 6).

In addition, there was a potent correlation between the reduced expression of the *MDM2* gene and a better prognosis across a wide variety of brain tissue subtypes, such as molecular, AJCC, Dukes, grade, and histological subtypes (Figure 7). There must be a way to figure out which gene is effectively responsible for the changes in the expression of and chances of survival in a specific cancer; that gene could be a potential biomarker for earlier cancer prognosis [53]. There is a relationship between the survival rate and the validation of possible biomarkers [54]. It is hypothesized that the *MDM2* gene could serve as a marker and inhibiter of tumors for the reduced expression of human breast, prostate, and gastric cancers [8,55]. Additionally, there is a positive link between *MDM2* overexpression and a poor prognosis for LGG cancer. Furthermore, the survival plots from multiple databases demonstrated that *MDM2* expression might play a role in LGG development and prognosis. The Pearson correlation coefficient and co-expression coefficients were used to evaluate the activity of the *MDM2* gene. In this study, the UALCAN and GEPIA databases were examined in order to find the genes that were positively linked with *MDM2* in LGG tissues. The cBioPortal interaction network was constructed using 20 associated genes from the website (Figure 8) and Gene MANIA, where Pearson CC > 0.47 was significant. Both databases estimated that RP11-61102.3 and YEATS4 had the highest Pearson CC value with the *MDM2* gene, which was corroborated by both databases. The interaction indicated 77.64 percent co-expression, indicating that the connected genes are expressed at similar levels in related conditions, and 3.63 percent co-localization, indicating that the correlated genes are expressed in a similar tissue or cell as the other related genes (Supplementary Figure S1). Gene MANIA was also used to evaluate another interaction network, and the network was constructed using the “automatically selected weighting approach” that was based on biologically processed data (Supplementary Figure S2) and, hence, 20 genes that interacted with each other were linked to a variety of cancers. MTBP has the possible prognostic characteristics of LGG [56]. *CDKN2A* has the potential to be the main target for the treatment of a number of human malignancies [57]. Moreover, *TP53* and *MDM4* have strong prognostic characteristics for the progression of brain cancer [58,59].

The in silico drug design approach has been becoming increasingly popular in recent times because of its proven potential to accelerate the discovery of safe and effective new medications [60,61,62]. This is accomplished through the evaluation of the outcomes of pharmacophore screening, molecular docking, analysis of post-docking interaction, molecular dynamic simulation (MDS), and prediction of noble and effective drug compounds against a higher range of diseases in a computer-simulated environment [63,64,65]. In silico drug design can be used to speed up the development of quality drugs by evaluating the outcomes of these processes [14,49]. Three of the four phytochemicals used in this study were effectively chosen to be lead compounds, while the fourth was used as a controlled drug for the purposes of this investigation. Along with conducting “fact checks” and providing secondary complementary judgments on high-performance assays, ADMET profiling is a cost-effective technique for significantly reducing the costs of drug development [66,67,68]. These four phytochemicals’ pharmacokinetic features were determined with the help of online servers from the pkCSM pharmacokinetics and Swiss ADME organizations, as indicated in (Table 2) [69,70,71]. As part of the study’s pharmacokinetic components, Lipinski’s five-rule pharmacophore properties (molecular weight, rotational bond count, logP value, hydrogen bond donors and acceptors, and degree of infringement) were evaluated for all selected ligands using Lipinski’s five-rule approach (violation level) [72,73,74]. All the selected natural bioactive phytochemicals possessed better human intestinal absorption, better BBB value, AMES toxicity, no carcinogenicity, and no hepatotoxicity for both the human and mice research model, except for the selected control compound for our research study [75,76,77].

Molecular docking is a technique for predicting the interactions between molecules under conditions with the most stringent compositional confirmation and the lowest binding affinity conceivable [78,79]. Using the Maestro application, which employs site-specific super molecular docking to assign a potential binding score, the drug candidates with the most significant and stable binding score were identified. Three natural bioactive compounds and a placebo were chosen for a molecular docking investigation with the p53-MDM2 protein (PDB ID: 1RV1). The control medicine, Temozolomide (PubChem CID 5394), was reported to have a docking affinity of −5.0 Kcal/mol. Among the natural bioactive compounds that were chosen, Taxifolin had the highest binding affinity of −10.0. This is the case when Galangin was −7.4 and (-)-Epicatechin was −8.8, as represented by Table 3. In the subsequent stage, Ligplot+ (Version 2.2), an excellent investigational tool that typically runs via the Java interface, was used to analyze the 2D protein–ligand interaction visualization. In addition, the docked receptor–ligand interactions were visualized using the Development Studio Visualizer tool v19.1.0.18287 (BIOVIA), a sophisticated visualizer tool for drug discovery (Figure 9 and Figure 10).

Molecular dynamic simulation (MDS) can help in the development of noble medications and performance data by providing insight into biomolecular interactions and the interface between the arrangement and activity of proteins [45,80], which was also conducted in our study using the YASARA software package. The simulation tool’s trajectory has also been used to flawlessly examine the root mean square deviation (RMSD), root mean square fluctuation (RMSF), the radius of gyration (Rg), the number of hydrogen bonds, and the surface area of the molecule that is accessible to solvent (SASA). It was determined how reliable the p53-MDM2 protein structure was by measuring the RMSD value of its protein backbone; a smaller number suggests more stable molecules. Our findings demonstrated that the RMSD values of protein–ligand interactions are adequate, with mean values of 2 (the lowest value for CAP is roughly 0.8, with maximum values of 3), indicating a more favorable docking location and no structural disruption between the two molecules (Figure 11A). The RMSF provides a quantitative measure of average protein fluctuations relative to some fixed point of reference, and RMSF graphs illustrate how these fluctuations are indicative of changes at the residue level. The RMSF of the c-alpha atoms can be seen illustrated in Figure 11B.

The total number of intermolecular hydrogen bonds developed between the macromolecules and their ligands was counted to determine the conformational stability, and the protein-ligands (-)-Epicatechin and Taxifolin were found to generate the most of these bonds, with strong conformational stability of 190 and 188, respectively. These protein-ligands are more conformationally stable than the control compound (Figure 11C). Using the simulation trajectories, the solvent-accessible surface area (SASA) of the protein-ligands was also measured to find out how the size of the drug-like molecules changed along the simulation trajectories [81]. Because of the structure’s instability, which places hydrophobic amino acid residues in close proximity to the water molecule, one of the highest SASA values is attributable to this factor [82]. The SASA result from the MDS trajectory revealed that Galangin exhibited the highest SASA values (6000 Å2); however, (-)-Epicatechin, Taxifolin, and Temozolomide (control) also had greater SASA values than what Galangin exhibited (Figure 11D).

In addition, Rg is a distance measurement between the protein’s center of mass and its terminus, and so it indicates how great that distance is. Consequently, this metric evaluates the compactness of the protein molecule and offers additional information regarding the folding features of the protein [83]. Furthermore, slackpacking is denoted by a higher Rg value, and compact packing is denoted by a lower Rg value (Kousar et al., 2020). The Rg values are summarized in (Figure 11E), with (-)-Epicatechin having the highest matching capacity (26.4) when compared to the reference or control ligand compound (Temozolomide, 26.5). The ligand compounds, Taxifolin and Galangin, are also compact, and numerical affinity values are reported in both cases (28.45). The weaker binding affinity was determined in quercetin and genistein (26.2). The protein–ligand complexes’ binding free energies were displayed by the Analysis of Molecular Mechanics Poisson-Boltzmann Surface Area (MMPBSA). In this case, all of the natural bioactive compounds chosen had more of the potential binding free energy value than the control drug compound (Figure 11F).

There was insufficient clinical evidence to substantiate LGG treatment by targeting the p53-MDM2 pathway loop in our current in silico analysis. This in silico study examined a novel method of treating LGG and found that more wet lab and clinical tests are required to confirm the efficacy of these drug-like compounds that target the MDM2 protein. Therefore, after the evaluation of their anticancer potential both the in vitro and in vivo research animals, these potentially bioactive phytochemicals may be exploited as an alternative therapeutic option for the human brain lower-grade glioma (LGG) cancer treatment.

## 4. Materials and Methods

### 4.1. MDM2 Gene Expression in Brain Cancer Research

Different types of online-based databases, namely, Oncomine (https://www.oncomine.org/, acessed on 21 July 2022), UALCAN (http://ualcan.path.uab.edu/, accessed on 21 July 2022), GENT2 (http://gent2.appex.kr/gent2/, accessed on 21 July 2022), and GEPIA2 (http://gepia2.cancer-pku.cn/#index, accessed on 21 July 2022) have been used to evaluate the mRNA transcription. They are all publicly available online interactive platform with different malignance assessments compared to the control at different cancers. Among all the datasets, Oncomine is currently the largest database of cancer microarray data of oncogenes in the world, with information about mining phases containing 715 independent datasets and 86.733 examples [84]. *MDM2* gene expression was compared and related in diseased and normal individuals where the *p*-value was 0.0001 and fold changes value was 2. On the UALCAN website, researchers looked at the expression of the targeted genes, methylation analysis, pan-cancer viewpoint, survival, and correlation data of the *MDM2* gene in brain cancer [85]. The levels of DNA methylation in the promoter region of the *MDM2* gene were also collected from the UALCAN data repository based on several criteria. The GENT2 database of Gene Expression was used for collecting *MDM2* gene expression data across 72 pairs of tissues [86]. Using the Gene Expression Profiling Interactive Analysis (GEPIA 2) database, the mRNA expression of the *MDM2* gene, in addition to its expression in cancer stages utilizing TCGA data in COAD, were explored further [87]. A gene-specific comparative examination of several malignant development patterns using a standard handling technique includes approximately 8587 normal and 9736 cancer samples.

### 4.2. Determination Copy Number Alterations and Mutation of MDM2 Gene

A multidisciplinary cancer genomic dataset such as “cBioPortal” (https://www.cbioportal.org/, accessed on 24 July 2022) possesses 308 cancer research datasets in this open-source research platform [88,89]. It analyzes the recurrence of mutations and other genetic abnormalities using data from over 5000 tumor samples and atomic characterization of cell lines and cancer tissues from various cancer investigations. We have also used this database to investigate mutations and variations in copy numbers in brain cancer for the *MDM2* gene.

### 4.3. Survival Data Analysis

Numerous virtual screening tools, notably GENT2, GEPIA, OncoLnc, and UALCAN were applied to identify the survival data of brain cancer patients according to alteration of time change against the measurement of *MDM2* gene. The survival condition of the diverse cancer subtypes was accurately determined via the GENT2 online server, where the result analysis comparison among 1146 samples and analyzed the 5 major subcategories, including the histology, grade, Dukes stage, AJCC stage, and molecular subtype. GEPIA (http://gepia.cancer-pku.cn/, accessed on 22 December 2021) is an online-based database that evaluates overall survival (OS) and disease-free survival (DFS) statistics [90]. A logrank test that is based on gene expression is utilized in order to evaluate the outcome when a particular form of cancer is diagnosed [91]. Survival analysis was also performed using the Onco-Lnc database (http://www.oncolnc.org/, accessed on 22 December 2021). It examines the expression of 21 cancers’ mRNA, miRNA, and lncRNA to determine the survival of 8647 patients [92]. In addition, the *MDM2* gene’s survival was examined using the UALCAN database. (http://ualcan.path.uab.edu/index.html, accessed on 22 December 2021). TCGA patient survival data were compared to Kaplan–Meier survival data, and then OS graphs were created and supplied [85].

### 4.4. Analysis of Correlation and Interaction Networks

For understanding better gene expression, it is necessary to categorize the target gene’s related genes. It was performed by searching associated genes with *MDM2* gene on the GEPIA and UALCAN databases. The GEPIA was used to identify the genes in TCGA LGG cancer that were comparable. The UALCAN database was used to find genes that are positively linked with *MDM2* in LGG cancer, and correlated or comparable genes were entered into the GeneMANIA website to assess the correlation between the two individual proteins. On both online sites, Pearson CC (correlation coefficients) > 0.47 was considered significant, and correlated or analogous genes were placed into the Gene MANIA website to evaluate the protein’s association. Gene MANIA (https://genemania.org/, accessed on 24 July 2022) is a web-based database that shows the connection between a set of genes and the genes that are entered. Genetic and protein relationships, co-localization, co-expression, pathway, and the similarity of protein domain are all included in the database [86]. The Gene MANIA server was utilized to estimate the *MDM2* gene’s protein–protein relationship to express the gene’s link with the listed genes.

### 4.5. Compounds Library Preparation and ADMET Screening for Selection of the Lead Compounds

To identify the anti-brain cancer activity, we constructed the phytochemical library. In our research study, we have selected 200 flavonoid compounds from diverse published scientific research articles based on their anticancer properties. Following the chemical properties analysis, 150 compounds have been chosen for future investigation. After that, we performed ADMET analysis to predict the pharmacokinetic features of test ligands, such as absorption, distribution, metabolism, excretion, and toxicity. To determine the ADME parameters, all 200 ligands were evaluated using the Swiss ADME (http://www.swissadme.ch/, accessed on 25 July 2022), which can be found here. Following this, toxicity profiling was performed on 200 ligands, of which only three did not break the Lipinski rule of violation. The web-based tool pkCSM (http://biosig.unimelb.edu.au/pkcsm/prediction, accessed on 25 July 2022) was implemented to find out how toxic the possible ligands were. It was decided to do additional computational investigations and analysis on three compounds.

### 4.6. Retrieval and Preparation of Compounds

From the PubChem database (https://pubchem.ncbi.nlm.nih.gov/, accessed on 26 July 2022), a spatial data file (SDF) containing the tertiary structure of the selected potentially bioactive aromatic compounds as well as the control drug were retrieved. Every ligand’s structure was prepared using the LigPrep application of Schrödinger software (https://www.schrodinger.com/products/maestro, accessed on 26 July 2022). The minimization process is carried out with the help of the OPLS3e force field and the Epik ionizer at a standard pH range of 7.0 to (+/−) 2.0, with a maximum of 32 conformers for each structure and an RMSD of 1.0.

### 4.7. Protein Retrieval and Preparation

The PDB format of the MDM2 protein structure (PDB ID: 1RV1) was retrieved from the Protein Data Bank (https://www.rcsb.org/, accessed on 27 July 2022). The Protein Preparation Wizard 12.5 of Maestro Application (Schrödinger 2020-3 Schrödinger, LLC, New York, NY, USA, 2020) was used for the preparation of the protein after it had been downloaded. The allocated bond orders, the chemical compound database (CCD), the insertion of hydrogens, the formation of disulfide bonds, the formation of zero-order bonds to metals, the completion of any missing side chains and loops utilizing Prime have been selected. In addition to utilizing the fixed cap termini and removing waters beyond 5 from heat groups, we also made use of Epik (Schrodinger Release version 2020-3), which was employed to generate heat states with pH 7.0 ± 2.0. The H-bond was identified in PROPKA at a pH level of 7.0, and the deterioration was capped at an RMSD of 0.30 by reducing the coverage of heavy atoms with the help of the refine tab and the OPLS3e force field. 

### 4.8. Active Site Prediction and Generation of Receptor Grid

An application known as SiteMap (Schrödinger Release 2021-2: SiteMap, Schrödinger, LLC, New York, NY, USA, 2021) was used, which can effectively identify the potential binding sites on proteins for small-molecule ligands by site map generation along with the evaluation of protein’s crystal and co-crystal structures. It pinpoints and ranks the sites on a protein’s surface that are most suited to bind with an external ligand. The current SiteMap facility in Maestro expands the original SiteMap facility that was previously available (named hapmap) [41,93]. Site mapping works similarly to Goodford’s GRID method, precisely in the original approach [94,95]. Three steps are involved in the SiteMap evaluation; in the first, the locations are initially defined by a grid, and then the dots are classified into subsets based on several parameters. In the second stage, grid mapping is used to create files that can be used to visualize the maps. Finally, the value of properties is determined, and locations are recorded in Maestro format. Every phase is completed by executing an impact job. For the purposes of molecular docking research, the optimal binding pocket was selected based on having a higher Site Score and DScore.

The Site Index is computed by doing a weighted average calculation on a number of different features:

SiteScore = 0.0733 sqrt(n) + 0.6688 e − 0.20 p.

In this equation, n represents the number of site points, which is capped at 100, e is the enclosure score, and p is the hydrophilic score, which is fixed at 1.0 in order to restrict the impact of hydrophilicity in charged and strongly polar sites [93,96,97].

Dscore and SiteScore are both calculated based on the same properties, although Dscore uses various coefficients and percentages:

DScore = 0.094 sqrt(n) + 0.60 e − 0.324 p.

There are no bounds on how high a hydrophilic score can be for Dscore. One of the criteria that differentiates “easy” and “druggable” targets from “difficult” and “undruggable” ones is this aspect of the target [93]. Different and even sometimes contradictory requirements necessitate the employment of distinct functions to identify binding sites and categorize drug consumption. It has been discovered that ligands with nanomolar and even sub-nanomolar affinities have been found for the PTP1B phosphate pocket [98,99]. However, they share some similarities with pharmaceutical drugs in terms of charge configuration, as these compounds are more closely related to those found in natural phosphate substrates and, hence, lack the drug-like properties typically associated with pharmaceuticals [49,100]. Even though SiteMap should identify a site with such high ligand interaction, the sitemap score should not consider it druggable.

### 4.9. Site Specific Super Molecular Docking

Maestro (Schrödinger Release 2021-2: Maestro, Schrödinger, LLC, New York, NY, USA, 2020-3.) was applied to XP program (extra precision) site-specific super molecular docking. After the completion of XP (extra precision), molecular docking by Maestro Tool (https://www.schrodinger.com/products/maestro, accessed on 26 July 2022) of Schrödinger Suite Software (https://www.schrodinger.com/, accessed on 26 July 2022), every protein–ligand complex structure in PDB format was taken from the docked post-viewing file for post-docking visualization, investigation of non-bond interactions, and evaluation of hydrophobicity and bioactivity.

### 4.10. Post-Docking Protein-Ligands Interactions Visualization

In order to investigate the interaction among the proteins and ligands, we implemented Ligplot+ version 2.2 to examine the polar and hydrophobic (non-bonding) interactions between protein–ligand complexes. This visualizing tool worked well owing to the java interface platform (Java SE Runtime Environment 8u271), which allowed only the combined PDB files that were retrieved from the Maestro application after molecular docking analysis [86,101]. The visualizer software Discovery Studio (http://media.accelrys.com/downloads/visualizer/45/DS45Client.exe, accessed on 26 July 2022) 64-bit application was used to perform post-docking visualization on the complex structures assessment. Additionally, the Discovery studio visualizer also evaluated the polar and non-polar interactions [102,103].

### 4.11. Molecular Dynamics Simulation (MDS)

The protein–ligand complexes were evaluated using 100 ns MD simulations to determine how consistently the chosen candidate compounds bind to the specified protein active site (AS) [104,105]. In the YASARA dynamics software program (version 4.3.13), molecular dynamics simulation research was conducted by using the AMBER14 force field. The MDS operation was conducted in a high-configured computer (Intel corei9, Nvidia RTX 3090 GPU, NVIDIA Corporation, Santa Clara, CA, USA) with windows operating system (OS). The complexes were subjected to preliminary cleaning, optimization, and hydrogen bond interaction management. Cubic simulation cells with periodic boundary conditions were employed alongside the TIP3P solvation model [106]. The temperature of the simulated system was 310 K, the pH was 7.4, and the salt concentration (NaCl) was 0.9% by weight [107]. The initial step in the process of minimizing the amount of energy used involved applying methods with the steepest gradient and an approach called simulated annealing (5000 cycles). Through the use of particle mesh Ewald techniques, long-range electrostatic interactions with a cutoff radius of 8 Å were computed [108,109]. The simulation systems were set up with a time step of 2.5 fs [107]. After each 100 ps of simulation time, the trajectories were saved. In order to investigate the RMSD, RMSF, Rg, SASA, and hydrogen bonding, the simulations trajectories were extended for 100 ns while adhering to constant pressure and the Berendsen thermostat.

Based on this, the YASARA trajectories were used in the computation of the binding free energy using the MM-PBSA methods. A higher positive energy value indicates a higher grade of energy [110,111]. The equation that was applied for the computations in order to determine the binding free energy was as follows:

Binding Energy = EpotRecept + EsolvRecept + EpotLigand + EsolvLigand − EpotComplex − EsolvComplex

All molecular dynamic simulation screenshots were generated using the premium YASARA software. According to the trajectory performance, the RMSD, RMSF, protein-ligand contacts (P-L), and the hydrogen bond interactions were conducted to measure whether or not the complex structure of protein and ligand could be maintained over time [112,113].

The RMSD in MD simulations is the average distance an atom moves in a given time interval with respect to a fixed time point [45,114]. To begin, the root-mean-square deviation (RMSD) of the protein’s structural atoms (C, backbone, side chain, and heavier particles) is calculated. Next, the RMSD of the protein’s fit ligand atoms over all time points is aligned and compared to the reference time point (in our study 100 ns). The RMSD of a periodic MD simulation with period *x* can be found with the application of the following equation (Equation (1)).

(1) $${\mathrm{RMSD}}_{x}=\sqrt{\frac{1}{N}}\;\sum_{i=1}^{N}(r\prime i\;(t_{x}))\;-\;r_{i}\;(t_{\mathrm{ref}}){)}^{2}$$

Here, *N* indicates the total number of atoms that were picked, *t_ref_* stands for the reference time, and *r*′ describes the location of the atoms that were chosen in system *x* after the point of the reference system has been superimposed on it.

The RMSF has mostly been utilized for detecting and tracking local variations in the conformational structure of protein complexes [115]. Calculating the RMSF of an MD simulation of a protein with the given number of residues and using the continuity equation (Equation (2)) is one possible way to do it.

(2) $${\mathrm{RMSF}}_{i}=\sqrt{\frac{1}{T}}\;\sum_{t=1}^{T}<(r\prime i(t))\;-\;r_{i}\;(t_{\mathrm{ref}}){)}^{2}>$$

Here, *T* generally denotes the trajectory time over which the RMSF is calculated; *t_ref_* is the reference; *r*′ denotes the position of the selected atoms in residue *i* after transposing on the reference frame, and angle bracket (< >) indicates the mean of the square distances covered over the selected atoms in the residues.

## 5. Conclusions

In this study, the aim was to precisely check the elevated level of *MDM2* gene in LGG disease compared to the standard samples and based on a variety of clinicopathological properties. Consequently, several integrative bioinformatics web-based tools were applied to publicly accessible datasets, revealing that *MDM2* may be an effective prognostic biomarker for this disease to determine the frequency of promoter methylation varies dramatically across the various phases of LGG. The level of methylation was greater in tumor samples compared to the normal or standard samples. The *MDM2* gene’s prognostic significance for LGG was determined using a KM plotter from multiple websites, including the ULCAN, Onco-Lnc, GENT2, and GEPIA; therefore, the survival plots from multiple databases demonstrated that *MDM2* expression might play a role in LGG development and prognosis. So, it is a major prognostic biomarker and acts as an alternative therapeutic site for the control of the advanced level of brain cancer. Additionally, the virtual mediated ADMET screening was used to identify 3 phytocompounds out of 50 that were predicted to have the most drug-like features. According to the results of the molecular docking study, these ligand molecules have the maximum binding affinity against the targeted receptor. This result was comparable to that of the reference anti-brain cancer medicine, Temozolomide. These pharmacological analogues, including Taxifolin, (-)-epicatechin, and Galangin, maintained more persistent and compact connections with the residues in the active region of MDM2 protein. The MD simulation was performed to confirm that these molecules have the best activity toward the targeted receptor, and they showed more positive results. As a result, they may be capable of overcoming the developing of LGG cancer. After all, these flavonoid-based compounds have the potential to be used as alternative therapeutic agents in LGG; nevertheless, additional study is necessary to verify that they do not possess any harmful side effects.

## Figures and Tables

**Table 1:** Inheritance changes in *MDM2* gene configurations linked to brain cancer growth progression in lower-grade gliomas (LGGs) are described. In total, 28 mutations were found, 6 of which were duplicated.

Cancer Study	Sample Size	Protein Change	Mutation Type	Sample ID
Brain Lower-Grade Glioma (TCGA Firehose Legacy)	530	S304P	Missense	TCGA-FG-8185-01
Brain Lower-Grade Glioma (TCGA PanCancer Atlas)	514	S304P	Missense	TCGA-FG-8185-01
		V207E	Missense	TCGA-KT-A7W1-01
Glioma (MSK, Nature 2019)	91	N334K	Missense	Patient-19-CSF
		N334K	Missense	Patient-19-T
		A351V	Missense	Patient-34-CSF-VP
Glioma (MSKCC, Clin Cancer Res 2019)	1004	I195V	Missense	P-0010402-T01-IM5
		S235N	Missense	P-0003900-T01-IM5
		R332G	Missense	P-0008166-T01-IM5
		G462E	Missense	P-0000500-T01-IM3
		E210K	Missense	P-0003900-T01-IM5
		E263K	Missense	P-0003900-T01-IM5
		G183D	Missense	P-0004400-T01-IM5
		V207A	Missense	P-0013506-T01-IM5
		G449d	Missense	TRF047202
		I208T	Missense	P-0019164-T01-IM6
Merged Cohort of LGG and GBM (TCGA, Cell 2016)	1102	S304P	Missense	TCGA-FG-8185-01
		V207E	Missense	TCGA-KT-A7W1-01
Glioblastoma (TCGA, Cell 2013)	543	V94M	Missense	TCGA-06-0155-01
		X229_splice	Splice	TCGA-12-0618-01
Glioblastoma (TCGA, Nature 2008)	206	Y287H	Missense	TCGA-02-0085-01
Glioblastoma Multiforme (TCGA, Firehose Legacy)	604	V94M	Missense	TCGA-06-0155-01
		X229_splice	Splice	TCGA-12-0618-01
Glioblastoma Multiforme (TCGA, PanCancer Atlas)	592	D86Y	Missense	TCGA-06-2566-01
		S127F	Missense	TCGA-06-5416-01
		I303M	Missense	TCGA-19-5956-01
		MDM2 CACNA1C	Fusion	TCGA-06-A7TK-01
		CTDSP2-MDM2	Fusion	TCGA-06-5856-06

**Table 2:** ADMET properties of flavonoid compounds.

Compds	MW (g/mol)	HBA	HBD	Num rot.	ToPoSA (Å^2^)	Log P	B.S.	LD_50_	BBB	HpT	AT	MToD	ToC
Temozolomide (control)	194.15	5	1	1	108.17	−0.92	0.55	2.178	−1.142	yes	yes	1.226	0.153
Taxifolin	304.25	7	5	1	127.45	0.63	0.55	2.261	−0.725	no	no	0.345	−0.078
(-)-Epicatechin	290.27	6	5	1	110.38	0.85	0.55	2.428	−1.00	no	no	0.438	0.183
Galangin	270.24	5	3	1	90.90	1.99	0.55	2.450	−0.748	no	no	0.333	0.256

Molecular weight (MW); hydrogen bond acceptor (HBA); hydrogen bond donor (HBD); number of rotatable bonds (Num. rot.); topological polar surface area (ToPoSA); predicted octanol/water partition coefficient (Log P); bioavailability score (B.S.); oral rat acute toxicity (LD_50_); blood brain barrier (BBB); hepatotoxicity (HpT); AMES toxicity (AT); maximum tolerated dose for human (MToD); total clearance (ToC).

**Table 3:** Tabulated in docking score, molecular interactions among selected phytochemicals with the targeted receptor.

Compounds	Docking Score (Kcal/mol)	Amino Acid Participation in Bonding Interaction	
		Interaction of Hydrogen Bond	Interaction of Hydrophobic Bond
Temozolomide (Reference Drug)	−5.0	Gln59 (3.04 Å)	Phe55, Phe55, Lys51, Gly58
Imidazoline (The native ligand of 1RV1)	−2.5		Leu54, Phe55, Gly59
Taxifolin	−10.0	Gln59 (2.81 Å), Gln59 (3.01 Å)	Lys51, Phe55, Gly58, Lys51, Phe55, Gln59
(-)-Epicatechin	−8.8	Lys51 (2.89 Å)	Leu54, Phe55, Gln59, Phe55, Leu54, Lys51
Galangin	−7.4		Lys51, Phe55, Gln59, Phe55, Lys51, Leu54

## Data Availability

For any additional data files other than Supplementary Information, if required you can contact the corresponding authors.

## Supplementary Materials

The following supporting information can be downloaded at: https://www.mdpi.com/article/10.3390/molecules28072977/s1, Supplementary Table S1: A comparison of *MDM2* transcriptional level between LGG tumor subtypes and regular individuals was made using the Oncomine database. Supplementary Table S2: Comparative analysis between normal and cancerous tissues utilizing UALCAN database to determine the expression pattern differences between *MDM2* and other genes in brain lower-grade glioma (LGG) cancer. *p*-value in bold denotes statistically significant values. Supplementary Table S3: Based on several characteristics and a comparative analysis between normal and different stages of cancer, the promoter methylation level of *MDM2* in brain lower-grade glioma (LGG) cancer was determined using the UALCAN database. *p*-value in bold denotes statistically significant values. Supplementary Table S4: Positively correlated genres of MDM2 gene of brain lower-grade glioma (LGG) cancer. Supplementary Figure S1: *MDM2* and the positively linked genes of brain lower-grade glioma cancer (LGG) have a lot of interactions with each other. Supplementary Figure S2: The correlation network of *MDM2* with its effectively interacted gene has been represented based on its physical and genetic interconnections, co-expression, co-localization, interaction pathways, and estimated protein domains.

## Sample Availability

Samples of all the compounds used in this research study are available from the authors.

## References

[1] Hayman L., Chaudhry W.R., Revin V.V., Zhelev N., Bourdon J.C. (2019). What is the potential of p53 isoforms as a predictive biomarker in the treatment of cancer?. Expert Rev. Mol. Diagn..

[2] Chi S.W., Lee S.H., Kim D.H., Ahn M.J., Kim J.S., Woo J.Y., Torizawa T., Kainosho M., Han K.H. (2005). Structural details on mdm2-p53 interaction. J. Biol. Chem..

[3] Harris S.L., Levine A.J. (2005). The p53 pathway: Positive and negative feedback loops. Oncogene.

[4] Zilani M.N.H., Islam M.A., Biswas P., Anisuzzman M., Hossain H., Shilpi J.A., Hasan M.N., Hossain M.G. (2021). Metabolite profiling, anti-inflammatory, analgesic potentials of edible herb Colocasia gigantea and molecular docking study against COX-II enzyme. J. Ethnopharmacol..

[5] Bouchet B.P., Caron de Fromentel C., Puisieux A., Galmarini C.M. (2006). p53 as a target for anti-cancer drug development. Crit. Rev. Oncol. Hematol..

[6] Paul P., Biswas P., Dey D., Saikat A.S.M., Islam M.A., Sohel M., Hossain R., Mamun A.A., Rahman M.A., Hasan M.N. (2021). Exhaustive Plant Profile of “Dimocarpus longan Lour” with Significant Phytomedicinal Properties: A Literature Based-Review. Processes.

[7] Nayak S.K., Panesar P.S., Kumar H. (2009). p53-Induced apoptosis and inhibitors of p53. Curr. Med. Chem..

[8] Rahman M.A., Rahman M.D.H., Hossain M.S., Biswas P., Islam R., Uddin M.J., Rahman M.H., Rhim H. (2020). Molecular Insights into the Multifunctional Role of Natural Compounds: Autophagy Modulation and Cancer Prevention. Biomedicines.

[9] Wang W., Hu Y. (2012). Small molecule agents targeting the p53-MDM2 pathway for cancer therapy. Med. Res. Rev..

[10] Zhang Q., Zeng S.X., Lu H. (2014). Targeting p53-MDM2-MDMX loop for cancer therapy. Subcell. Biochem..

[11] Shvarts A., Steegenga W.T., Riteco N., van Laar T., Dekker P., Bazuine M., van Ham R.C., van der Houven van Oordt W., Hateboer G., van der Eb A.J. (1996). MDMX: A novel p53-binding protein with some functional properties of MDM2. EMBO J..

[12] Rahman M.A., Rahman M.H., Biswas P., Hossain M.S., Islam R., Hannan M.A., Uddin M.J., Rhim H. (2020). Potential Therapeutic Role of Phytochemicals to Mitigate Mitochondrial Dysfunctions in Alzheimer’s Disease. Antioxidants.

[13] Fu T., Min H., Xu Y., Chen J., Li G. (2012). Molecular dynamic simulation insights into the normal state and restoration of p53 function. Int. J. Mol. Sci..

[14] Arefin A., Ismail Ema T., Islam T., Hossen S., Islam T., Al Azad S., Uddin Badal N., Islam A., Biswas P., Alam N.U. (2021). Target specificity of selective bioactive compounds in blocking α-dystroglycan receptor to suppress Lassa virus infection: An in silico approach. J. Biomed. Res..

[15] Bibi S., Hasan M.M., Biswas P., Shkodina A., Shah M.A., Shah G.M., Khan A., Al-Harrasi A., Khan H., Akkol E.K., Daglia M. (2022). Chapter 7—Phytonutrients in the management of lipids metabolism. The Role of Phytonutrients in Metabolic Disorders.

[16] Pinzi L., Caporuscio F., Rastelli G. (2018). Selection of protein conformations for structure-based polypharmacology studies. Drug Discov. Today.

[17] Pei D., Zhang Y., Zheng J. (2012). Regulation of p53: A collaboration between Mdm2 and Mdmx. Oncotarget.

[18] Biswas P., Dey D., Rahman A., Islam M.A., Susmi T.F., Kaium M.A., Hasan M.N., Rahman M.D.H., Mahmud S., Saleh M.A. (2021). Analysis of SYK Gene as a Prognostic Biomarker and Suggested Potential Bioactive Phytochemicals as an Alternative Therapeutic Option for Colorectal Cancer: An In-Silico Pharmaco-Informatics Investigation. J. Pers. Med..

[19] Dey D., Hossain R., Biswas P., Paul P., Islam M.A., Ema T.I., Gain B.K., Hasan M.M., Bibi S., Islam M.T. (2022). Amentoflavone derivatives significantly act towards the main protease (3CL(PRO)/M(PRO)) of SARS-CoV-2: In silico admet profiling, molecular docking, molecular dynamics simulation, network pharmacology. Mol. Divers..

[20] Liu E.T., Lemberger T. (2007). Higher order structure in the cancer transcriptome and systems medicine. Mol. Syst. Biol..

[21] McMurray H.R., Sampson E.R., Compitello G., Kinsey C., Newman L., Smith B., Chen S.R., Klebanov L., Salzman P., Yakovlev A. (2008). Synergistic response to oncogenic mutations defines gene class critical to cancer phenotype. Nature.

[22] Ozaki T., Nakagawara A. (2011). Role of p53 in Cell Death and Human Cancers. Cancers.

[23] Laird P.W., Jaenisch R. (1996). The role of DNA methylation in cancer genetic and epigenetics. Annu. Rev. Genet..

[24] Esteller M. (2003). Cancer epigenetics: DNA methylation and chromatin alterations in human cancer. Adv. Exp. Med. Biol..

[25] Schiebe M., Ohneseit P., Hoffmann W., Meyermann R., Rodemann H.P., Bamberg M. (2000). Analysis of mdm2 and p53 gene alterations in glioblastomas and its correlation with clinical factors. J. Neurooncol..

[26] Choi S.R., Lee M. (2022). Estimating the Prognosis of Low-Grade Glioma with Gene Attention Using Multi-Omics and Multi-Modal Schemes. Biology.

[27] Rayan A., Raiyn J., Falah M. (2017). Nature is the best source of anticancer drugs: Indexing natural products for their anticancer bioactivity. PLoS ONE.

[28] Khan M.S., Mehmood B., Yousafi Q., Bibi S., Fazal S., Saleem S., Sajid M.W., Ihsan A., Azhar M., Kamal M.A. (2021). Molecular Docking Studies Reveal Rhein from rhubarb (Rheum rhabarbarum) as a Putative Inhibitor of ATP-binding Cassette Super-family G member 2. Med. Chem..

[29] Fridlender M., Kapulnik Y., Koltai H. (2015). Plant derived substances with anti-cancer activity: From folklore to practice. Front. Plant Sci..

[30] Caputi L., Franke J., Farrow S.C., Chung K., Payne R.M.E., Nguyen T.D., Dang T.T., Soares Teto Carqueijeiro I., Koudounas K., Dugé de Bernonville T. (2018). Missing enzymes in the biosynthesis of the anticancer drug vinblastine in Madagascar periwinkle. Science.

[31] Slichenmyer W.J., Von Hoff D.D. (1991). Taxol: A new and effective anti-cancer drug. Anticancer Drugs.

[32] Brahmer J.R., Ettinger D.S. (1998). The Role of Topotecan in the Treatment of Small Cell Lung Cancer. Oncologist.

[33] Ashraf M.A., Sayed S., Bello M., Hussain N., Chando R.K., Alam S., Hasan M.K. (2022). CDK4 as a phytochemical based anticancer drug target. Inform. Med. Unlocked.

[34] Singh J., Sangwan N., Chauhan A., Avti P.K. (2022). Integrative network and computational simulation of clinical and genomic data for the identification of mutated EGFR in breast cancer patients for therapeutic targeting using purine analogues. Mol. Simul..

[35] Malik S. (2017). Biotechnology and Production of Anti-Cancer Compounds.

[36] Hamedi A., Bayat M., Asemani Y., Amirghofran Z. (2022). A review of potential anti-cancer properties of some selected medicinal plants grown in Iran. J. Herb. Med..

[37] Ferdausi N., Islam S., Rimti F.H., Quayum S.T., Arshad E.M., Ibnat A., Islam T., Arefin A., Ema T.I., Biswas P. (2022). Point-specific interactions of isovitexin with the neighboring amino acid residues of the hACE2 receptor as a targeted therapeutic agent in suppressing the SARS-CoV-2 influx mechanism. J. Adv. Vet. Anim. Res..

[38] Siddiqui A.J., Jahan S., Singh R., Saxena J., Ashraf S.A., Khan A., Choudhary R.K., Balakrishnan S., Badraoui R., Bardakci F. (2022). Plants in Anticancer Drug Discovery: From Molecular Mechanism to Chemoprevention. Biomed Res. Int..

[39] Hasan A., Biswas P., Bondhon T.A., Jannat K., Paul T.K., Paul A.K., Jahan R., Nissapatorn V., Mahboob T., Wilairatana P. (2022). Can Artemisia herba-alba Be Useful for Managing COVID-19 and Comorbidities?. Molecules.

[40] Hossain R., Dey D., Biswas P., Paul P., Ahmed S.Z., Khan A.A., Ema T.I., Islam M.T. (2022). Chlorophytum borivilianum (Musli) and Cimicifuga racemosa (Black Cohosh). Herbs, Shrubs, and Trees of Potential Medicinal Benefits.

[41] Halgren T. (2007). New method for fast and accurate binding-site identification and analysis. Chem. Biol. Drug Des..

[42] Fu C., Deng S., Koneski I., Awad M.M., Akram Z., Matinlinna J., Pichika M.R., Daood U., Fawzy A.S. (2020). Multiscale in-vitro analysis of photo-activated riboflavin incorporated in an experimental universal adhesive. J. Mech. Behav. Biomed. Mater..

[43] Friesner R.A., Banks J.L., Murphy R.B., Halgren T.A., Klicic J.J., Mainz D.T., Repasky M.P., Knoll E.H., Shelley M., Perry J.K. (2004). Glide: A new approach for rapid, accurate docking and scoring. 1. Method and assessment of docking accuracy. J. Med. Chem..

[44] Ojo O.A., Ojo A.B., Okolie C., Nwakama M.C., Iyobhebhe M., Evbuomwan I.O., Nwonuma C.O., Maimako R.F., Adegboyega A.E., Taiwo O.A. (2021). Deciphering the Interactions of Bioactive Compounds in Selected Traditional Medicinal Plants against Alzheimer’s Diseases via Pharmacophore Modeling, Auto-QSAR, and Molecular Docking Approaches. Molecules.

[45] Bharadwaj S., Dubey A., Yadava U., Mishra S.K., Kang S.G., Dwivedi V.D. (2021). Exploration of natural compounds with anti-SARS-CoV-2 activity via inhibition of SARS-CoV-2 Mpro. Brief. Bioinform..

[46] Opo F., Rahman M.M., Ahammad F., Ahmed I., Bhuiyan M.A., Asiri A.M. (2021). Structure based pharmacophore modeling, virtual screening, molecular docking and ADMET approaches for identification of natural anti-cancer agents targeting XIAP protein. Sci. Rep..

[47] Sastry G.M., Adzhigirey M., Day T., Annabhimoju R., Sherman W. (2013). Protein and ligand preparation: Parameters, protocols, and influence on virtual screening enrichments. J. Comput. Aided Mol. Des..

[48] Carr-Wilkinson J., O’Toole K., Wood K.M., Challen C.C., Baker A.G., Board J.R., Evans L., Cole M., Cheung N.K., Boos J. (2010). High Frequency of p53/MDM2/p14ARF Pathway Abnormalities in Relapsed Neuroblastoma. Clin. Cancer Res..

[49] Biswas P., Dey D., Biswas P.K., Rahaman T.I., Saha S., Parvez A., Khan D.A., Lily N.J., Saha K., Sohel M. (2022). A Comprehensive Analysis and Anti-Cancer Activities of Quercetin in ROS-Mediated Cancer and Cancer Stem Cells. Int. J. Mol. Sci..

[50] Onel K., Cordon-Cardo C. (2004). MDM2 and prognosis. Mol. Cancer Res..

[51] Deben C., Deschoolmeester V., Lardon F., Rolfo C., Pauwels P. (2016). TP53 and MDM2 genetic alterations in non-small cell lung cancer: Evaluating their prognostic and predictive value. Crit. Rev. Oncol. Hematol..

[52] Beroukhim R., Mermel C.H., Porter D., Wei G., Raychaudhuri S., Donovan J., Barretina J., Boehm J.S., Dobson J., Urashima M. (2010). The landscape of somatic copy-number alteration across human cancers. Nature.

[53] Zeng Z., Yang Y., Qing C., Hu Z., Huang Y., Zhou C., Li D., Jiang Y. (2020). Distinct expression and prognostic value of members of SMAD family in non-small cell lung cancer. Medicine.

[54] Gyorffy B., Lánczky A., Szállási Z. (2012). Implementing an online tool for genome-wide validation of survival-associated biomarkers in ovarian-cancer using microarray data from 1287 patients. Endocr. Relat. Cancer.

[55] Wang W., Chen Z., Jin J., Long Z., Liu X., Cai H., Zhou Y., Huang H., Wang Y. (2017). MDM2 binding protein as a predictor of metastasis and a novel prognostic biomarker in patients with gastric cancer. Oncol. Lett..

[56] Song Y., Zhang L., Jiang Y., Hu T., Zhang D., Qiao Q., Wang R., Wang M., Han S. (2019). MTBP regulates cell survival and therapeutic sensitivity in TP53 wildtype glioblastomas. Theranostics.

[57] Inoue R., Moghaddam K.A., Ranasinghe M., Saeki Y., Chiocca E.A., Wade-Martins R. (2004). Infectious delivery of the 132 kb CDKN2A/CDKN2B genomic DNA region results in correctly spliced gene expression and growth suppression in glioma cells. Gene Ther..

[58] Riemenschneider M.J., Büschges R., Wolter M., Reifenberger J., Boström J., Kraus J.A., Schlegel U., Reifenberger G. (1999). Amplification and overexpression of the MDM4 (MDMX) gene from 1q32 in a subset of malignant gliomas without TP53 mutation or MDM2 amplification. Cancer Res..

[59] Xiong Y., Zhang Y., Xiong S., Williams-Villalobo A.E. (2020). A Glance of p53 Functions in Brain Development, Neural Stem Cells, and Brain Cancer. Biology.

[60] Bibi S., Sakata K. (2016). Current Status of Computer-Aided Drug Design for Type 2 Diabetes. Curr. Comput. Aided Drug Des..

[61] Dey D., Biswas P., Paul P., Mahmud S., Ema T.I., Khan A.A., Ahmed S.Z., Hasan M.M., Saikat A.S.M., Fatema B. (2022). Natural flavonoids effectively block the CD81 receptor of hepatocytes and inhibit HCV infection: A computational drug development approach. Mol. Divers..

[62] Sohel M., Biswas P., Al Amin M., Hossain M.A., Sultana H., Dey D., Aktar S., Setu A., Khan M.S., Paul P. (2022). Genistein, a Potential Phytochemical against Breast Cancer Treatment-Insight into the Molecular Mechanisms. Processes.

[63] Ahmed H., Mahmud A.R., Siddiquee M.F.R., Shahriar A., Biswas P., Shimul M.E.K., Ahmed S.Z., Ema T.I., Rahman N., Khan M.A. (2022). Role of T cells in cancer immunotherapy: Opportunities and challenges. Cancer Pathog. Ther..

[64] Al Azad S., Ahmed S., Biswas P., Mia M.A.R., Farjana M., Arshe F.A., Mily S.J., Ankhi A.B., Shaikat M.M., Sultana10 S. (2022). Quantitative analysis of the factors influencing IDA and TSH downregulation in correlation to the fluctuation of activated vitamin D3 in women. J. Adv. Biotechnol. Exp. Ther..

[65] Paul P.K., Al Azad S., Rahman M.H., Farjana M., Uddin M.R., Dey D., Mahmud S., Ema T.I., Biswas P., Anjum M. (2022). Catabolic profiling of selective enzymes in the saccharification of non-food lignocellulose parts of biomass into functional edible sugars and bioenergy: An in silico bioprospecting. J. Adv. Vet. Anim. Res..

[66] Mannhold R., Kubinyi H., Folkers G. (2012). Pharmacokinetics and Metabolism in Drug Design.

[67] Notari R.E. (1973). Pharmacokinetics and molecular modification: Implications in drug design and evaluation. J. Pharm. Sci..

[68] Biswas P., Hasan M.M., Dey D., Dos Santos Costa A.C., Polash S.A., Bibi S., Ferdous N., Kaium M.A., Rahman M.D.H., Jeet F.K. (2021). Candidate antiviral drugs for COVID-19 and their environmental implications: A comprehensive analysis. Environ. Sci. Pollut. Res. Int..

[69] Matin M.M., Roshid M.H., Bhattacharjee S.C., Azad A.K. (2020). PASS predication, antiviral, in vitro Antimicrobial, and ADMET studies of rhamnopyranoside esters. Med. Res. Arch..

[70] Rahman M.S., Zilani M.N.H., Islam M.A., Hasan M.M., Islam M.M., Yasmin F., Biswas P., Hirashima A., Rahman M.A., Hasan M.N. (2021). In Vivo Neuropharmacological Potential of Gomphandra tetrandra (Wall.) Sleumer and In-Silico Study against β-Amyloid Precursor Protein. Processes.

[71] Al Saber M., Biswas P., Dey D., Kaium M.A., Islam M.A., Tripty M.I.A., Rahman M.H., Rahaman T.I., Biswas M.Y., Paul P. (2022). A Comprehensive Review of Recent Advancements in Cancer Immunotherapy and Generation of CAR T Cell by CRISPR-Cas9. Processes.

[72] Munshi M., Zilani M.N.H., Islam M.A., Biswas P., Das A., Afroz F., Hasan M.N. (2022). Novel compounds from endophytic fungi of Ceriops decandra inhibit breast cancer cell growth through estrogen receptor alpha in in-silico study. Inform. Med. Unlocked.

[73] Aziz S., Bibi S., Hasan M.M., Biswas P., Ali M.I., Bilal M., Chopra H., Mukerjee N., Maitra S. (2023). A review on influence of biochar amendment on soil processes and environmental remediation. Biotechnol. Genet. Eng. Rev..

[74] Biswas P., Polash S.A., Dey D., Kaium M.A., Mahmud A.R., Yasmin F., Baral S.K., Islam M.A., Rahaman T.I., Abdullah A. (2023). Advanced implications of nanotechnology in disease control and environmental perspectives. Biomed. Pharmacother..

[75] Hasan M.M., Zilani M.N.H., Akter S., Nasrin P., Shajib G.M.A., Islam M.A., Biswas P., Mahmud S., Saleh M.A., Hasan M.N. (2022). UHPLC-Q/Orbitrap/MS based chemical fingerprinting and hepatoprotective potential of a medicinal plant, Morinda angustifolia Roxb. S. Afr. J. Bot..

[76] Sarker M.T., Saha S., Biswas P., Islam M.T., Sheikh M.A., Hasan M.N., Islam N., Rabbe M.M.I., Rafi M.O. (2022). Identification of blood-based inflammatory biomarkers for the early-stage detection of acute myocardial infarction. Netw. Model. Anal. Health Inform. Bioinform..

[77] Singh R., Khalid M., Batra N., Biswas P., Singh L., Bhatti R. (2023). Exploring the Anticonvulsant Activity of Aqueous Extracts of *Ficus benjamina* L. Figs in Experimentally Induced Convulsions. J. Chem..

[78] Dey D., Paul P.K., Al Azad S., Al Mazid M.F., Khan A.M., Sharif M.A., Rahman M.H. (2021). Molecular optimization, docking, and dynamic simulation profiling of selective aromatic phytochemical ligands in blocking the SARS-CoV-2 S protein attachment to ACE2 receptor: An in silico approach of targeted drug designing. J. Adv. Vet. Anim. Res..

[79] Dipta D., Tanzila Ismail E., Partha BISWAS S.A., Shoeba ISLAM U.R.R., FIROZ M., AHMED S.Z., Salauddin A., RAHMAN A., AFRIN10 S., MAHEDI10 R.A.J.F.A.S.E. (2021). Antiviral effects of bacteriocin against animal-to-human transmittable mutated SARS-CoV-2: A systematic review. Front. Agric. Sci. Eng..

[80] Aljahdali M.O., Molla M.H.R., Ahammad F. (2021). Compounds Identified from Marine Mangrove Plant (*Avicennia alba*) as Potential Antiviral Drug Candidates against WDSV, an In-Silico Approach. Mar. Drugs.

[81] Elfiky A.A., Elshemey W.M. (2018). Molecular dynamics simulation revealed binding of nucleotide inhibitors to ZIKV polymerase over 444 nanoseconds. J. Med. Virol..

[82] Mahmud S., Rahman E., Nain Z., Billah M., Karmakar S., Mohanto S.C., Paul G.K., Amin A., Acharjee U.K., Saleh M.A. (2021). Computational discovery of plant-based inhibitors against human carbonic anhydrase IX and molecular dynamics simulation. J. Biomol. Struct. Dyn..

[83] Alamri M.A., Altharawi A., Alabbas A.B., Alossaimi M.A., Alqahtani S.M. (2020). Structure-based virtual screening and molecular dynamics of phytochemicals derived from Saudi medicinal plants to identify potential COVID-19 therapeutics. Arab. J. Chem..

[84] Rhodes D.R., Kalyana-Sundaram S., Mahavisno V., Varambally R., Yu J., Briggs B.B., Barrette T.R., Anstet M.J., Kincead-Beal C., Kulkarni P. (2007). Oncomine 3.0: Genes, pathways, and networks in a collection of 18,000 cancer gene expression profiles. Neoplasia.

[85] Chandrashekar D.S., Bashel B., Balasubramanya S.A.H., Creighton C.J., Ponce-Rodriguez I., Chakravarthi B., Varambally S. (2017). UALCAN: A Portal for Facilitating Tumor Subgroup Gene Expression and Survival Analyses. Neoplasia.

[86] Park S.J., Yoon B.H., Kim S.K., Kim S.Y. (2019). GENT2: An updated gene expression database for normal and tumor tissues. BMC Med. Genom..

[87] Tang Z., Kang B., Li C., Chen T., Zhang Z. (2019). GEPIA2: An enhanced web server for large-scale expression profiling and interactive analysis. Nucleic Acids Res..

[88] Cerami E., Gao J., Dogrusoz U., Gross B.E., Sumer S.O., Aksoy B.A., Jacobsen A., Byrne C.J., Heuer M.L., Larsson E. (2012). The cBio cancer genomics portal: An open platform for exploring multidimensional cancer genomics data. Cancer Discov..

[89] Gao J., Aksoy B.A., Dogrusoz U., Dresdner G., Gross B., Sumer S.O., Sun Y., Jacobsen A., Sinha R., Larsson E. (2013). Integrative analysis of complex cancer genomics and clinical profiles using the cBioPortal. Sci. Signal..

[90] Tang Z., Li C., Kang B., Gao G., Li C., Zhang Z. (2017). GEPIA: A web server for cancer and normal gene expression profiling and interactive analyses. Nucleic Acids Res..

[91] Anaya J. (2016). OncoLnc: Linking TCGA survival data to mRNAs, miRNAs, and lncRNAs. PeerJ Comput. Sci..

[92] Warde-Farley D., Donaldson S.L., Comes O., Zuberi K., Badrawi R., Chao P., Franz M., Grouios C., Kazi F., Lopes C.T. (2010). The GeneMANIA prediction server: Biological network integration for gene prioritization and predicting gene function. Nucleic Acids Res..

[93] Halgren T.A. (2009). Identifying and characterizing binding sites and assessing druggability. J. Chem. Inf. Model..

[94] Goodford P.J. (1985). A computational procedure for determining energetically favorable binding sites on biologically important macromolecules. J. Med. Chem..

[95] Weber A.E., Halgren T.A., Doyle J.J., Lynch R.J., Siegl P.K., Parsons W.H., Greenlee W.J., Patchett A.A. (1991). Design and synthesis of P2-P1′-linked macrocyclic human renin inhibitors. J. Med. Chem..

[96] Khan R.A., Hossain R., Siyadatpanah A., Al-Khafaji K., Khalipha A.B.R., Dey D., Asha U.H., Biswas P., Saikat A.S.M., Chenari H.A. (2021). Diterpenes/Diterpenoids and Their Derivatives as Potential Bioactive Leads against Dengue Virus: A Computational and Network Pharmacology Study. Molecules.

[97] Baral S.K., Biswas P., Kaium M.A., Islam M.A., Dey D., Al Saber M., Rahaman T.I., Emran T.B., Hasan M.N., Jeong M.K. (2022). A Comprehensive Discussion in Vaginal Cancer Based on Mechanisms, Treatments, Risk Factors and Prevention. Front. Oncol..

[98] Thérien M., Skorey K., Zamboni R., Li C.S., Lau C.K., LeRiche T., Linh Truong V., Waddleton D., Ramachandran C. (2004). Synthesis of a novel peptidic photoaffinity probe for the PTP-1B enzyme. Bioorg. Med. Chem. Lett..

[99] Morshed A., Al Azad S., Mia M.A.R., Uddin M.F., Ema T.I., Yeasin R.B., Srishti S.A., Sarker P., Aurthi R.Y., Jamil F. (2022). Oncoinformatic screening of the gene clusters involved in the HER2-positive breast cancer formation along with the in silico pharmacodynamic profiling of selective long-chain omega-3 fatty acids as the metastatic antagonists. Mol. Divers..

[100] Bibi S., Sakata K. (2017). An Integrated Computational Approach for Plant-Based Protein Tyrosine Phosphatase Non-Receptor Type 1 Inhibitors. Curr. Comput. Aided Drug. Des..

[101] Rahman M.D.H., Biswas P., Dey D., Hannan M.A., Sahabuddin M., Araf Y., Kwon Y., Emran T.B., Ali M.S., Uddin M.J. (2022). An In-Silico Identification of Potential Flavonoids against Kidney Fibrosis Targeting TGFβR-1. Life.

[102] Studio D. (2008). Discovery Studio.

[103] Saleem U., Iman S., Ahmad B., Shah M.A., Bibi S., Alqarni M., Khan M.S., Shah G.M., Khan H., Alhasani R.H. (2023). Antidepressant activity of phytochemicals of Mangifera indica seeds assisted by integrated computational analysis. Metab. Brain Dis..

[104] Hoffman J.M., Margolis K.G. (2020). Building community in the gut: A role for mucosal serotonin. Nat. Rev. Gastroenterol. Hepatol..

[105] Bibi S., Hasan M.M., Wang Y.B., Papadakos S.P., Yu H. (2022). Cordycepin as a Promising Inhibitor of SARS-CoV-2 RNA Dependent RNA Polymerase (RdRp). Curr. Med. Chem..

[106] Harrach M.F., Drossel B. (2014). Structure and dynamics of TIP3P, TIP4P, and TIP5P water near smooth and atomistic walls of different hydroaffinity. J. Chem. Phys..

[107] Krieger E., Vriend G. (2015). New ways to boost molecular dynamics simulations. J. Comput. Chem..

[108] Essmann U., Perera L., Berkowitz M.L., Darden T., Lee H., Pedersen L.G. (1995). A smooth particle mesh Ewald method. J. Chem. Phys..

[109] Krieger E., Nielsen J.E., Spronk C.A., Vriend G. (2006). Fast empirical pKa prediction by Ewald summation. J. Mol. Graph. Model..

[110] Dash R., Ali M.C., Dash N., Azad M.A.K., Hosen S.M.Z., Hannan M.A., Moon I.S. (2019). Structural and Dynamic Characterizations Highlight the Deleterious Role of SULT1A1 R213H Polymorphism in Substrate Binding. Int. J. Mol. Sci..

[111] Islam M.A., Zilani M.N.H., Biswas P., Khan D.A., Rahman M.H., Nahid R., Nahar N., Samad A., Ahammad F., Hasan M.N. (2022). Evaluation of in vitro and in silico anti-inflammatory potential of some selected medicinal plants of Bangladesh against cyclooxygenase-II enzyme. J. Ethnopharmacol..

[112] Bibi S., Khan M.S., El-Kafrawy S.A., Alandijany T.A., El-Daly M.M., Yousafi Q., Fatima D., Faizo A.A., Bajrai L.H., Azhar E.I. (2022). Virtual screening and molecular dynamics simulation analysis of Forsythoside A as a plant-derived inhibitor of SARS-CoV-2 3CLpro. Saudi Pharm. J..

[113] Dey D., Hasan M.M., Biswas P., Papadakos S.P., Rayan R.A., Tasnim S., Bilal M., Islam M.J., Arshe F.A., Arshad E.M. (2022). Investigating the Anticancer Potential of Salvicine as a Modulator of Topoisomerase II and ROS Signaling Cascade. Front. Oncol..

[114] Biswas P., Hany Rumi O., Ahmed Khan D., Ahmed M.N., Nahar N., Jahan R., Hasan Zilani M.N., Paul T.K., Hasan A., Bondhon T.A. (2022). Evaluation of Melongosides as Potential Inhibitors of NS2B-NS3 Activator-Protease of Dengue Virus (Serotype 2) by Using Molecular Docking and Dynamics Simulation Approach. J. Trop. Med..

[115] Han Y., Wang Z., Ren J., Wei Z., Li J. (2021). Potential inhibitors for the novel coronavirus (SARS-CoV-2). Brief. Bioinform..

