# Supplementary material for: Study of MDM2 as Prognostic Biomarker in Brain-LGG Cancer and Bioactive Phytochemicals Inhibit the p53-MDM2 Pathway: A Computational Drug Development Approach
Source: Molecules. 2023 Mar 27;28(7):2977. doi: 10.3390/molecules28072977 (PMC10095937; doi:10.3390/molecules28072977)
Supplement: Supplementary file 1 [file molecules-28-02977-s001.zip › molecules-2154182-supplementary.pdf]

Supplementary Table S1: Transcription levels of *MDM2* gene between different subtypes of Brain Lower Grade Glioma (LGG) Cancer and normal individuals using the Oncomine database.

Dataset	Brain Cancer Subtype	*P*-value	Fold change	*t*-test	Gene Rank (%)
**Murat Brain**	Glioblastoma (80)	1.22E-8	2.523	7.331	3%
**Bredel Brain 2**	Glioblastoma (27)	6.85E-5	2.269	5.040	8%
**Watson Brain**	Meningioma (15)	0.131	1.740	1.357	28%
**Liang Brain**	Oligoastrocytoma (3)	0.026	1.417	2.811	7%
	Glioblastoma (30)	0.02	1.488	3.798	12%
**Lee Brain**	Glioblastoma (22)	7.67E-4	6.131	6.040	7%
**Rickman Brain**	Astrocytoma (45)	0.182	1.147	0.947	35%
**Pomeroy Brain**	Desmoplastic Medulloblastoma (14)	0.184	4.415	1.020	37%
	Malignant Glioma, NOS (10)	0.261	2.096	0.679	44%
	Atypical Terapoid/Rhabdoid Tumor (50	0.187	4.228	1.028	46%
	Classic Medulloblastoma (46)	0.401	1.468	0.272	52%
**Sun Brain**	Glioblastoma (81)	2.82E-8	1.674	5.873	11%
	Oligodendroglioma (50)	7.92E-4	1.284	3.288	17%
	Diffuse Astrocytoma (7)	0.040	1.396	1.904	21%
	Anaplastic Astrocytoma (19)	0.019	1.147	2.152	26%
**TCGA Brain**	Brain Glioblastoma (542)	1.65E-5	1.341	5.128	22%
	Glioblastoma (5)	0.297	1.145	0.573	395
**Shai Brain**	Oligodendroglioma (3)	0.120	1.078	1.430	32%
	Glioblastoma (27)	0.069	1.051	1.576	34%
	Astrocytoma (5)	0.549	-1.008	-0.127	56%

**Beroukhim Brain**	Primary Glioblastoma (106)	0.008	1.105	2.454	15%
	Secondary Glioblastoma (15)	0.048	1.028	1.727	23%
	Anaplastic Oligodendroglioma (7)	0.393	1.005	0.273	40%
	Anaplastic Astrocytoma (9)	0.851	-1.055	-1.110	82%
**French Brain**	Anaplastic Oligodendroglioma (23)	0.001	1.590	3.625	15%
	Anaplastic Oligodendroglioma (4)	0.090	2.202	1.702	31%
**Gutmann Brain**	Pilocytic Astrocytoma	0.274	1.700	0.694	38%
**TCGA Brain 2**	Brain Glioblastoma (582)	1.49E-13	1.128	7.469	26%
	Glioblastoma (29)	0.156	1.083	1.081	29%
	Brain Astrocytoma (63)	0.493	1.000	0.019	57%
	Oligoastrocytoma (54)	0.765	-1.005	-0.727	61%
	Brain Oligodendroglioma (89)	0.133	1.010	1.121	67%
	Malignant Glioma, NOS (14)	0.805	-1.022	-0.889	74%

Supplementary Table S2: Differential expression pattern analysis of *MDM2* in Brain Lower Grade Glioma (LGG) Cancer based on different variables and different stages where the comparison was made between normal vs different stages of cancer using UALCAN database. *P*-value in bold denotes statistically significant values.

Variables	Different stages	N	Comparisons	Statistical significance
**Patient's race**	Caucasian	474	Caucasian-vs- African American	8.138000E-01
	African- American	21	Caucasian-vs-Asian	2.419600E-01

	Asian	8	African American –vs- Asian	1.609420E-01
**Patients gender**	Male	285	Male-vs-female	9.919200E-01
	Female	229		
**Patient's age**	21-40 Yrs.	243	Age(21-40Yrs)-vs-Age(41-60Yrs)	3.551800E-02
	41-60 Yrs.	200	Age(21-40Yrs)-vs-Age(61-80Yrs)	1.855590E-01
	61-80 Yrs.	60	Age(21-40Yrs)-vs-Age(81-100Yrs)	N/A
	81-100 Yrs.	1	N/A	N/A
**Tumor grade**	Grade 2	248	Grade 2-vs-Grade 3	1.294290E-02
	Grade 3	265		
**Histological subtype**	Astrocytoma	194	Astrocytoma-vs-Oligoastrocytoma	1.494230E-01
	Oligoastrocytoma	130	Astrocytoma-vs-Oligodendroglioma	3.175000E-01
	Oligodendroglioma	191	Oligoastrocytoma-vs-Oligodendroglioma	8.741500E-03
**TP53 mutation status**	TP53-Mutant	230	TP53-Mutant-vs- TP53-NonMutant	1.524250E-03
	TP53-NonMutant	282		

Supplementary Table S3: Promoter methylation level of *MDM2* in Brain Lower Grade Glioma (LGG) Cancer based on different variables and in a comparison between normal vs different stages of cancer from UALCAN database. *P*-value in bold denotes statistically significant values**.**

Variables	Different stages	N	Comparisons	Statistical significance
**Patient's race**	Caucasian	474	Caucasian-vs- African American	8.839000E-01
	African- American	21	Caucasian-vs-Asian	5.540000E-01

	Asian	8	African American –vs- Asian	5.359600E-01
**Patients gender**	Male	285	Male-vs-female	9.695200E-01
	Female	230		
**Patient's age**	21-40 Yrs.	244	Age(21-40Yrs)-vs-Age(41-60Yrs)	8.842800E-01
	41-60 Yrs.	200	Age(21-40Yrs)-vs-Age(61-80Yrs)	9.494200E-01
	61-80 Yrs.	60	Age(21-40Yrs)-vs-Age(81-100Yrs)	N/A
	81-100 Yrs.	1	N/A	N/A
**TP53 mutation status**	TP53-Mutant	231	TP53-Mutant-vs- TP53-NonMutant	6.480800E-01
	TP53-NonMutant	282		

Supplementary Table S4: Positively correlated genes of *MDM2* genes responsible for the progression of Brain Lower Grade Glioma (LGG) Cancer.

**GEPIA**		**UALCAN**	
**Gene Symbol**	**PCC**	**Gene Symbol**	**PCC**
RP11-61102.3	0.86	YEATS4	0.92
RP11-61102.5	0.8	CNOT2	0.91
SLC35E3	0.6	CCT2	0.91
METTL1	0.58	FRS2	0.9
CDK4	0.55	PTPRB	0.89
NUP107	0.52	FAM119B	0.8

TSPAN31	0.52	RAB3IP	0.73
METTL21B	0.52	TSFM	0.71
AGAP2-AS1	0.48	METTL1	0.59
OS9	0.48	TSPAN31	0.58
		LOC100130776	0.52
		CDK4	0.49

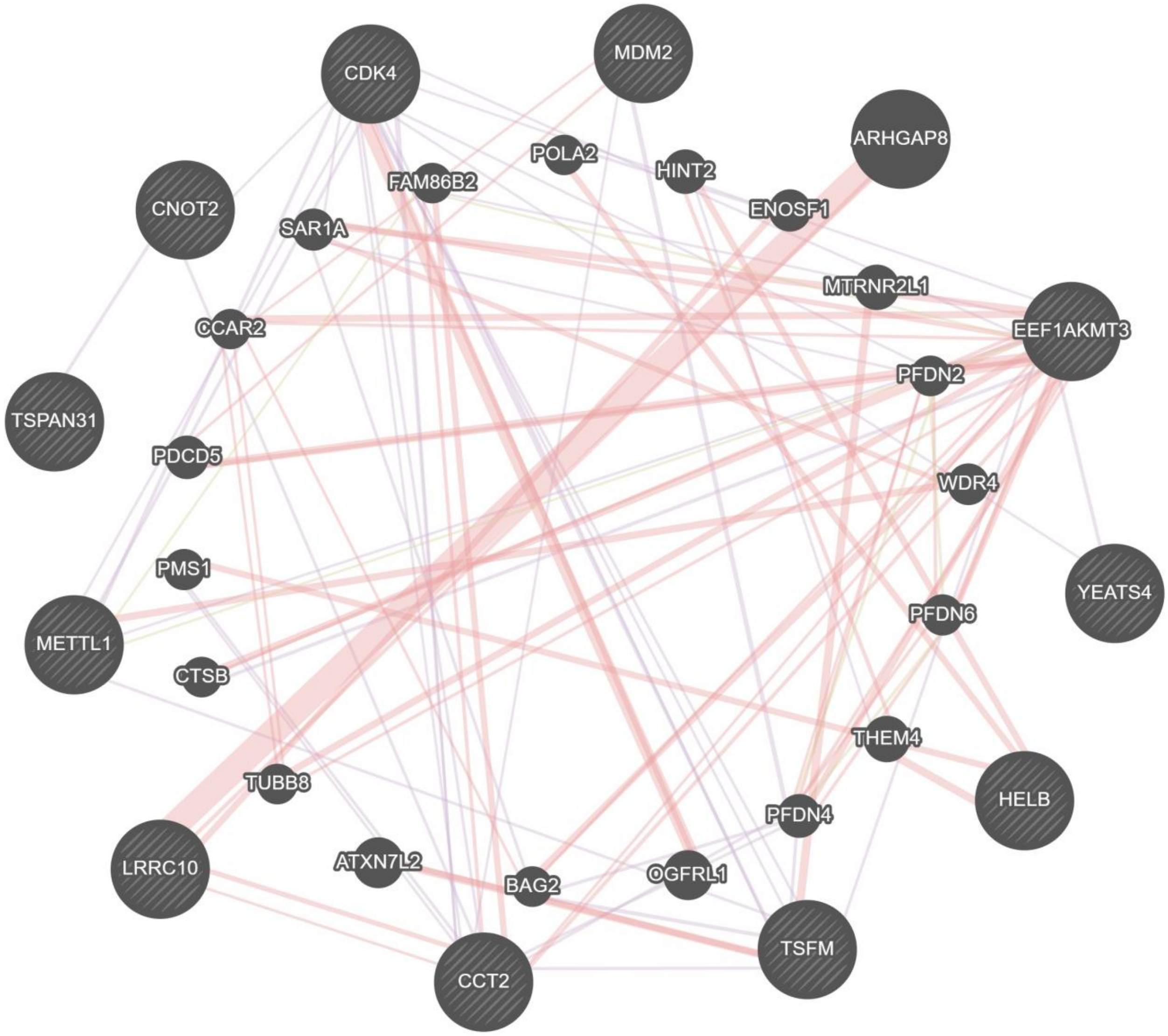

Supplementary Figure S1: The protein-protein interaction network of *MDM2* with the positively correlated genes of Brain Lower Grade Glioma Cancer (LGG).

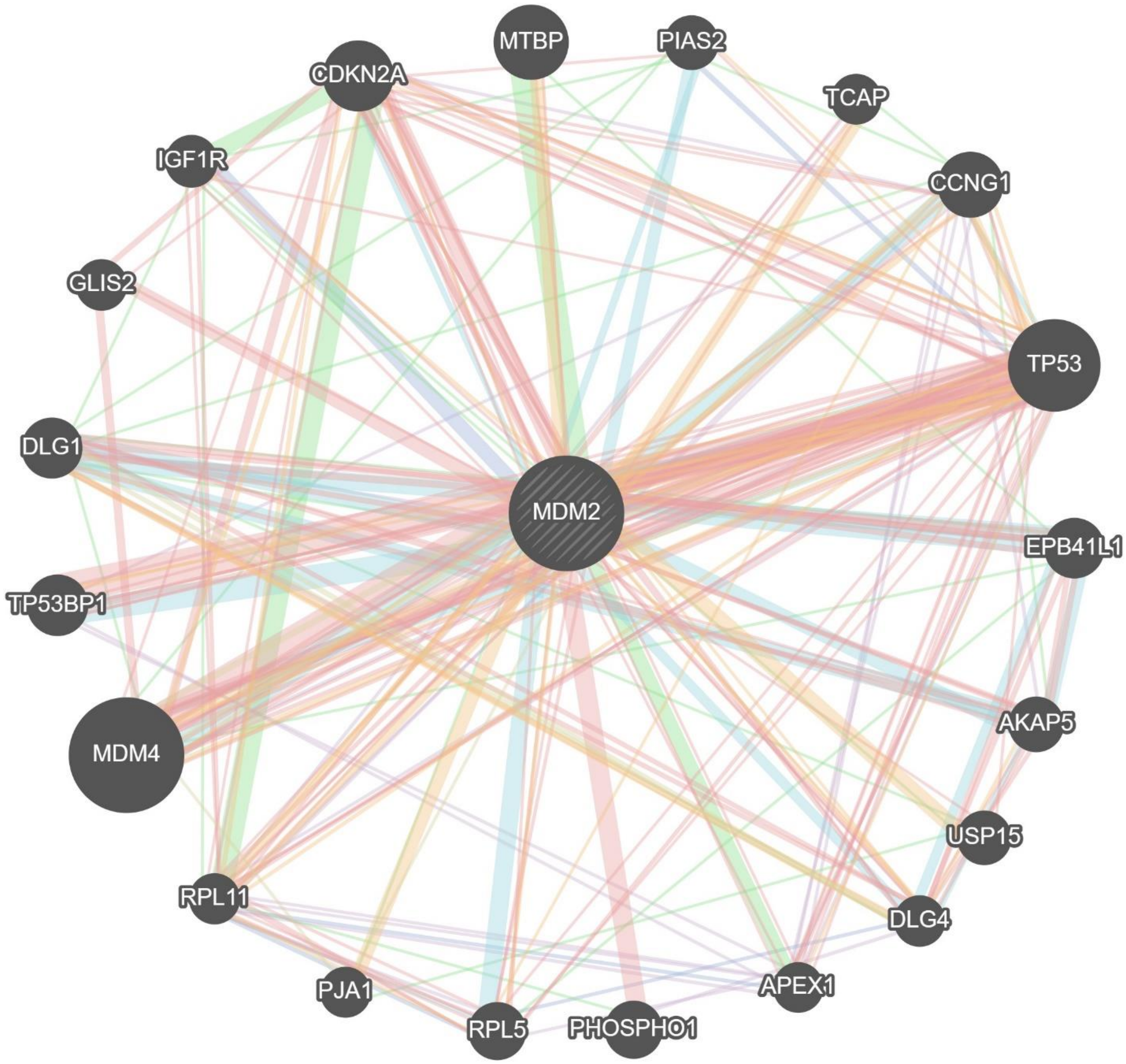

Supplementary Figure S2: The correlation network of *MDM2* with its effectively interacted gene have been represented based on its physical and genetic interconnections, co-expression, co-localization, interaction pathways and estimated protein domains.
